# Characterization of the Statistical Signatures of Micro-Movements Underlying Natural Gait Patterns in Children with Phelan McDermid Syndrome: Towards Precision-Phenotyping of Behavior in ASD

**DOI:** 10.3389/fnint.2016.00022

**Published:** 2016-06-27

**Authors:** Elizabeth B. Torres, Jillian Nguyen, Sejal Mistry, Caroline Whyatt, Vilelmini Kalampratsidou, Alexander Kolevzon

**Affiliations:** ^1^Department of Psychology, Computer Science, Rutgers Center for Cognitive Sciences and Computational Biomedicine Imaging and Modelling Center of Computer Science, Rutgers The State University of New JerseyNew Brunswick, NJ, USA; ^2^Graduate Program in Neuroscience, Rutgers The State University of New JerseyNew Brunswick, NJ, USA; ^3^Department of Mathematics, Rutgers The State University of New JerseyNew Brunswick, NJ, USA; ^4^Department of Psychology, Rutgers The State University of New JerseyNew Brunswick, NJ, USA; ^5^Department of Computer Science, Rutgers The State University of New JerseyNew Brunswick, NJ, USA; ^6^Psychiatry, Seaver Autism Center for Research and Treatment, Icahn School of Medicine at Mount SinaiNew York, NY, USA

**Keywords:** micro-movements, noise, PMS, Phelan-McDermid syndrome, stochastic signatures, gait, gamma distribution, precision phenotyping

## Abstract

**Background:** There is a critical need for precision phenotyping across neurodevelopmental disorders, especially in individuals who receive a clinical diagnosis of autism spectrum disorder (ASD). Phelan-McDermid deletion syndrome (PMS) is one such example, as it has a high penetrance of ASD. At present, no biometric characterization of the behavioral phenotype within PMS exists.

**Methods:** We introduce a data-type and statistical framework that permits the personalized profiling of naturalistic behaviors. Walking patterns were assessed in 30 participants (16 PMS, 3 idiopathic-ASD and 11 age- and sex-matched controls). Each individual's micro-movement signatures were recorded at 240 Hz. We empirically estimated the parameters of the continuous Gamma family of probability distributions and calculated their ranges. These estimated stochastic signatures were then mapped on the Gamma plane to obtain several statistical indexes for each child. To help visualize complex patterns across the cohort, we introduce new tools that enable the assessment of connectivity and modularity indexes across the peripheral network of rotational joints.

**Results:** Typical walking signatures are absent in all children with PMS as well as in the children with idiopathic-ASD (iASD). Underlying these patterns are atypical leg rotational acceleration signatures that render participants with PMS unstable with rotations that are much faster than controls. The median values of the estimated Gamma parameters serve as a cutoff to automatically separate children with PMS 5–7 years old from adolescents with PMS 12–16 years old, the former displaying more randomness and larger noise. The fluctuations in the arm's motions during the walking also have atypical statistics that separate males from females in PMS and show higher rates of noise accumulation in idiopathic ASD (iASD) children. Despite high heterogeneity, all iASD children have excess noise, a narrow range of probability-distribution shapes across the body joints and a distinct joint network connectivity pattern. Both PMS and iASD have systemic issues with noise in micro-motions across the body with specific signatures for each child that, as a cohort, selectively deviates from controls.

**Conclusions:** We provide a new methodology for precision behavioral phenotyping with the potential to use micro-movement output noise as a natural classifier of neurodevelopmental disorders of known etiology. This approach may help us better understand idiopathic neurodevelopmental disorders and personalize the assessments of natural movements in these populations.

## Introduction

Phelan–McDermid syndrome (PMS) is a complex neurodevelopmental disorder associated with efferent and afferent neurological abnormalities, believed to emerge from underlying impairments in synaptic transmission and synaptic plasticity (Phelan et al., 2001; Wilson et al., 2003). The origins of such problems can be traced back to heterozygous deletions of chromosome 22q13.3 (Durand et al., 2007; Moessner et al., 2007; Bonaglia et al., 2011), which encodes for the *SHANK3* gene. SHANK3 codes for a scaffold protein located at the post-synaptic density (PSD) of glutamatergic synapses. Specifically, they are important for the formation and stabilization of synapses, as they assemble glutamate receptors with their intracellular signaling apparatus and cytoskeleton at the PSD (Roussignol et al., 2005). Other *SHANK* genes in different locations of the genome also play a role in neural development. In neurons, *SHANK2* and *SHANK3* have a positive effect on the induction and maturation of dendritic spines, whereas *SHANK1* induces the enlargement of spine heads (Roussignol et al., 2005).

Research in *SHANK*-related disorders has primarily focused on neurons from the central nervous system, but we propose that similar disruptions of synaptic transmission and plasticity exist across the *peripheral* sensory and motor nerves in the nervous system. In principle, any of the *SHANK*-related disruptions could alter synaptic flow, increase synaptic noise throughout the periphery, and consequently compromise the re-afferent flow of peripheral sensory feedback that emerge from self-produced movements (von Holst and Mittelstaedt, 1950; Von Holst, 1954). Some evidence for peripheral disruption has been reported in *Shank3* mouse and rat models (Raab et al., 2010). These disruptions paired with cerebellar deficits in 22q13 deletion syndrome (Aldinger et al., 2013) could interfere with the formation and maturation of internal models for action (IMA) in this population. Paired with the hypotonic issues that the children present with at birth (Soorya et al., 2013), these disruptions may impact motor noise, compromise their corporeal self-awareness, and delay achievement of sensory-motor developmental milestones.

Disruptions within the peripheral nervous system from an early age may also impact the formation of body maps and frames of reference for motor action. These are critical ingredients for the central control of behaviors, as specified within the theoretical framework of IMA (Kawato and Wolpert, 1998; Wolpert et al., 1998). In particular, this framework indicates that, in order to compensate for synaptic transduction and transmission delays, central planning and control require forward estimation of impending action commands (and of their efference copy), as well as estimation of their sensory consequences. Evidence suggests that these processes take place in the primate brain (Mulliken et al., 2008; Andersen and Cui, 2009; Torres et al., 2013c), where afferent projections from the periphery are anatomically present (Prevosto et al., 2009, 2010, 2011). These afferent projections provide sensory feedback that may also include statistical regularities emerging from the continuous stream of self-produced movements [movements that are generated and controlled from the central nervous system (CNS)]. Indeed, disruptions in the peripheral afferent signals are known to impede movement (Cole, 1995; Stenneken et al., 2006; Balslev et al., 2007; Torres et al., 2014) (even when efferent output flow is intact), as well as alter the conscious recognition of one's own actions (Fourneret et al., 2002).

Self-produced movements under CNS control contain minute fluctuations (micro-movements) that may also serve as an additional source of peripheral afferent feedback. This putative source-component of afferent feedback can be statistically estimated (non-invasively), with millisecond time precision at the motor output level (Torres et al., 2013a, Wu et al., 2014). Recent research has profiled disruptions in the flow of micro-movements in children with a diagnosis of idiopathic autism spectrum disorder (iASD) (Torres et al., 2013a), thus calling for the investigation of the signatures of micro-movements in children with PMS who also receive an ASD diagnosis. PMS accounts for up to 2% of ASD cases (Leblond et al., 2014). As such, PMS may serve as a valid model to better understand similar manifestations in sub-groups of iASD (Betancur and Buxbaum, 2013). Indeed, it is likely that the sensory and motor issues associated with PMS (Battaglia, 2011; Soorya et al., 2013) interfere with social interactions and contribute to the ASD phenotype. The phenotype of PMS has been described using parent-report measures and subjective observational assessments, however, an objective profiling of sensory-motor patterns has not been developed to date. Detailed sensory-motor phenotyping in a single gene form of ASD may increase the likelihood of linking deficits in social behavior to physical sensory-motor disruptions caused by specific synaptic problems due to underlying genetic factors.

In this paper, we offer a new type of precision-phenotyping model of human behavior to initiate steps toward achieving Precision Psychiatry. The proposed model is based on the ***individualized*** statistical characterization of the stochastic signatures of micro-movements underlying the types of overt and covert movements that make up naturalistic behaviors in the social environment that the person (inevitably) shares with others. Overt movements are defined by explicit goals and are deliberately performed, whereas covert movements are driven by less obvious implicit goals and are highly automatic. Both movement classes coexist within a gradient of intentionality and contribute to the flow of motions along a continuum (Torres, 2012).

Our approach addresses the Research Domain Criteria (RDoC), a recent initiative of the National Institutes of Mental Health (NIMH), to “*Develop, for research purposes, new ways of classifying mental disorders based on dimensions of observable behavior and neurobiological measures.*” The aim of RDoC is to identify core features—some yet undiscovered or underutilized—that cut across research domains and that use rigorous scientific method (Insel, 2014). Specifically, RDoC addresses the lack of validity of the Diagnostic and Statistical Manual for Mental Disorders (DSM) [and the International Classification of Diseases (ICD)], but it is at present lacking a motor domain (Bernard and Mittal, 2015). In this sense, the micro-movements would enable a form of personalized precision phenotyping as part of the broader NIH's Precision Medicine initiative (Hawgood et al., 2015).

In the specific context of neurodevelopment, this new methodology could equally benefit disorders that result in social deficits and those that do not. We illustrate this framework using the gait patterns of 16 children with PMS in relation to 11 age- and sex-matched controls. Together, with novel analytical methods presented in this study, we provide new visualization tools to examine synchronous patterns of peripheral joints' coordination automatically detecting self-emerging synergies of co-articulated joints across the body. Since there is high penetrance of ASD in PMS (84%; Soorya et al., 2013), we also include three individuals with a diagnosis of iASD for reference. We report a pattern of foot rotations present in controls that is systematically absent in the children with PMS, and 2/3 of the children with iASD. We further unveil fundamental differences in joint rotation and coordination across the body between typically developing controls and children with PMS and iASD. In particular, we point at stochastic signatures of upper body and arm rotations during gait that separate all individuals by sex. Results are discussed within the context of the Research Domain Criteria (RDoC) framework, highlighting the ability of micro-movements-based dynamic biometrics to provide a new type of precision-phenotyping for human behaviors. This research approach is amenable to connect natural behaviors, particularly elements of body entrainment, joint attention, volitional control and other socio-motor features critical for social exchange, to underlying genetic information.

## Methods

This study took place at the Sensory Motor Integration Laboratory of Rutgers University. Participants with PMS were recruited and clinically assessed at the Seaver Autism Center for Research and Treatment at the Icahn School of Medicine at Mount Sinai, NYC. All participants and/or parents signed the consent form approved by the Rutgers University Institutional Review Board (IRB). The entire study protocol was approved by the Rutgers University IRB. Clinical records were obtained in compliance with the Health Insurance Portability and Accountability Act (HIPAA). The study conforms to the guidelines of the Helsinki Act for the use of human subjects in research.

## Subjects

There were 16 participants with PMS in this study, 9 females and 7 males, ranging from 5 to 15 years old. Eleven of the 16 children were previously described in the literature (Soorya et al., 2013; Kolevzon et al., 2014). Supplementary Tables 1, 2 provide demographic information. We note that three children reportedly wore ankle braces permanently to aid in walking. In such cases the braces were not removed to minimize the risk of falling, as per their parents' recommendations.

Control subjects (11) and subjects with iASD (3) also participated in the study. Controls included 5 females and 6 males, ranging from 5 to 19 years old, including one 19 year old professional athlete. The three male participants with iASD ranged from 10 to 12 years old. In addition to a diagnosis of ASD, one participant also had a diagnosis of ADHD and Tourette's syndrome. His inclusion served as an interesting point of comparison. Specifically, as there have been reports of comorbid ASD and Tourette's syndrome (Lawson-Yuen et al., 2008), this participant provides additional characterization of motor signatures across the broad spectrum of ASD.

## Interdisciplinary assessment

An interdisciplinary team evaluated patients using the following clinical evaluation tools (described in Soorya et al., 2013):

1. *Psychiatric evaluations* using the DSM-5 were conducted by board-certified child and adolescent psychiatrists at the first patient visit and focused on the assessment of ASD.
2. *Clinical genetics evaluations* and dysmorphology examinations were performed by clinical geneticists to assess growth, pubertal development, head size, craniofacial features, digits, extremities, chest, spine, skin, organ malformations (such as congenital heart or renal defects) and neurological abnormalities.
3. *Neurological examinations* were conducted by a pediatric neurologist to evaluate gross motor skills and gait, fine motor coordination, cranial nerves and deep tendon reflexes.
4. *Autism Diagnostic Observation Schedule-Toddler Module* (Luyster et al., 2009; Lord et al., 2012) is a direct semi-structured assessment that was used to assess the presence communication, reciprocal social interaction and repetitive, restricted behaviors. The ADOS- Toddler Module, intended for minimally verbal individuals with nonverbal age equivalents down to 12 months, was administered by a trained clinician to all patients in this sample.
5. *ADI-R* (Lord et al., 1994) is an investigator-based, semi-structured interview used to differentiate autistic disorder from non-autistic ID in individuals with a developmental age greater than 18 months old. It was administered by a trained clinician to parents or caregivers, making use of an algorithm that incorporates the DSM-IV criteria for diagnosis.
6. *Cognitive testing* was conducted by licensed clinical psychologists or doctoral students to provide estimates of cognitive functioning. The Mullen Scales of Early Learning (Mullen, 1995) were used on all participants, including children with chronological ages older than the standardization sample, to allow for flexible administration of items. Ratio intelligence quotients (IQs) were calculated using mental age estimates from the cognitive tests and used to provide an estimate of nonverbal IQ.
7. The *Vineland Adaptive Behavior Scale, Second Edition* (Sparrow et al., 1984) was used to evaluate independence in daily life skills, including communication, socialization and motor skills.

The Supplementary Table 1 lists these scores from all 16 children with PMS.

## Genetic testing

Chromosomal microarray analysis (CMA) or Sanger sequencing was used to confirm *SHANK3* deficiency in 16/16 patients with PMS due to deletions or mutations respectively.

## Experimental setup

**Figure 1:**
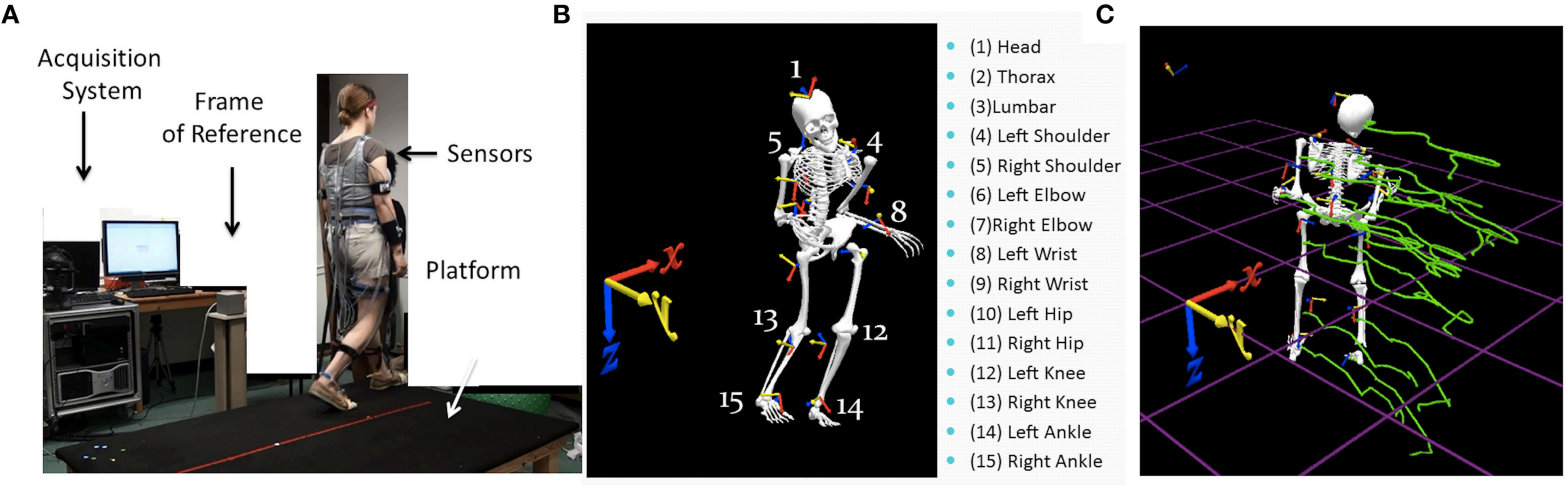
**Experimental set up and sample parameters. (A)** Gait platform where subjects paced back and forth and performed other naturalistic interactions during walking. The acquisition system, the wearable sensors affixed across the body, and the frame of reference used to obtain the kinematics from the movement trajectories are also shown. **(B)** Digitization of the full body using 15 electromagnetic sensors attached to body parts enumerated on the right and the orientation of the frame of reference. **(C)** Sample kinematic trajectories in green showing the positional changes over time as the person walks in one direction.

Since participants with PMS were unable to follow precise verbal instructions and perform decision-making tasks and/or experiments with a variety of cognitive loads, this study examined a naturalistic behavior that they were all able to perform—natural gait. All walking took place on a raised platform to cover the proper distances defining the weak electro-magnetic field created by the sensing system (Polhemus Liberty, Colchester, VT) recording at 240 Hz. The platform also ensures a systematic walking pattern, requiring turns and back-and-forth pacing, across all subjects (see Figure 1A).

## Analytical methods

We analyzed micro-movements underlying gait patterns for all participants. Micro-movements are minute fluctuations in overt and covert movements that are imperceptible to the naked eye, but quantifiable through kinematics and dynamic analyses of the motor output. They are present in the time-series of waveforms registering physiological signals from various systems including electroencephalographic activity, the various motions of the eyes, those of the full body, as well as autonomic signals from the heart, respiration, temperature, skin conductance, etc. In this work we focus on the motor output of body movements during gait. Motor outputs continuously flow in closed loop from the CNS to the periphery and back. We used full-body kinematics from time-series data of gait. A 30-min (minimum) period of walking was recorded for each child, which was repeated across a number of sessions, including pre- and post-treatment in a clinical trial with Insulin-Like Growth Factor-1 (ClinicalTrials.gov Identifier: NCT01525901). This paper focuses on the first (baseline) session.

### First layer of data

We first analyzed the kinematics layer of raw positional and orientation data along with their inherent variability unique to each individual. The raw displacement and angular rotational data are the outputs of the 15 sensors (Polhemus Liberty, Colchester, VT), 14 attached to the body and one used for calibration and digitization purposes (Figure 1B). Data were collected using the Motion Monitor Interface (InnSport, Chicago, IL). Various in-home programmed filters, as well as filters built into this data collection system, provided ways to remove instrumentation noise so as to focus on the data that may be physiologically relevant. All data in this set were treated identically before the second layer of stochastic signatures described below was estimated.

**Figure 2:**
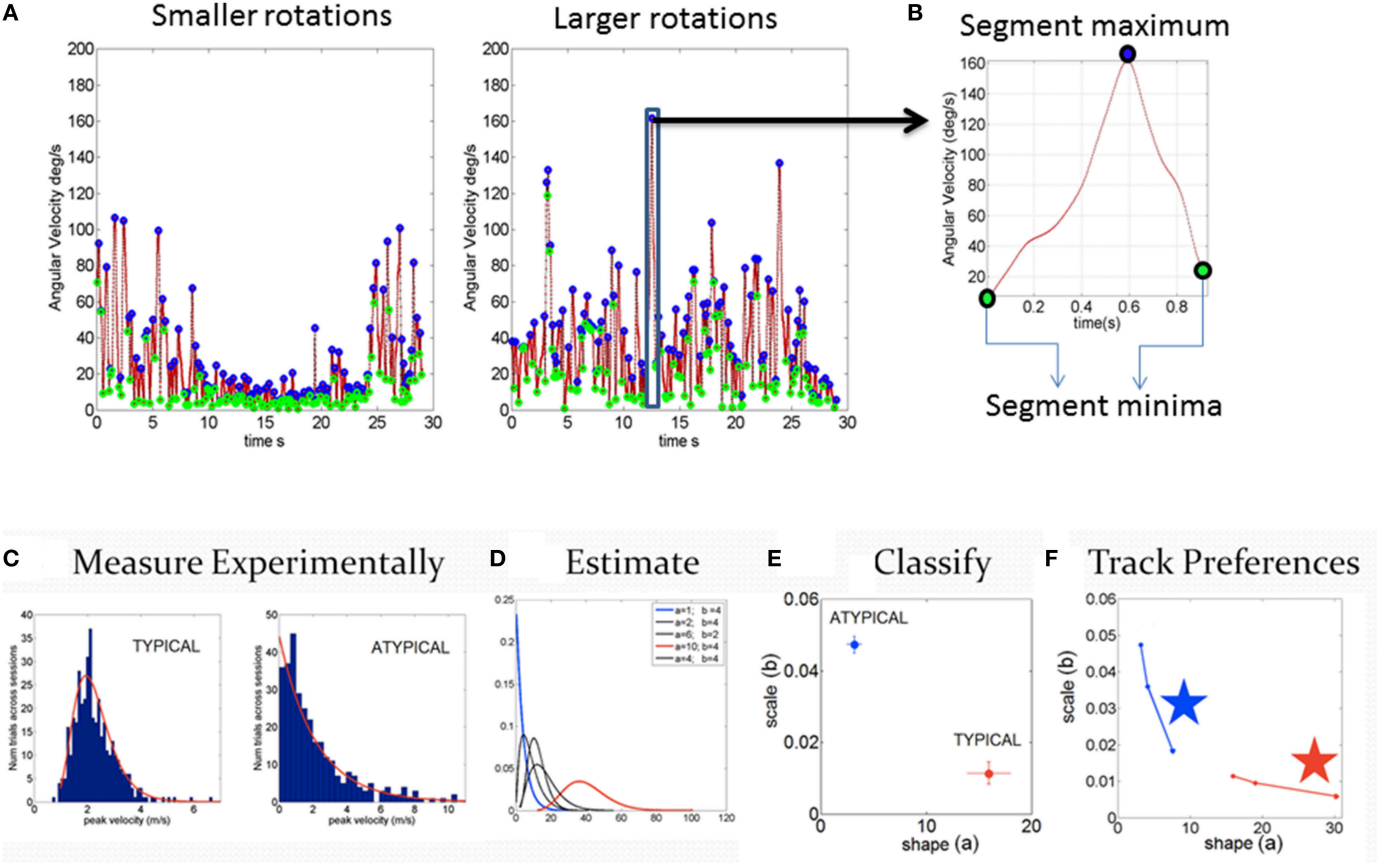
**Steps to harness and analyze kinematics data. (A)** Samples of smaller and larger angular velocity values (rate of change of rotation at the joints, deg/sec) continuously registered and shown for 7200 frames (30 s)-segments. **(B)** Zooming into the time series of angular speed data to obtain the normalized peak velocity index (described in the text) and trace a time series of a normalized waveform. **(C–F)** Steps to obtain statistical estimates from empirical data. **(C)** *Step1* measure the parameters continuously in naturalistic movements. Gather the parameter of interest (e.g., peak linear velocity or peak angular velocity, etc. obtained from the first order change of position over time for each sensor position across the body) into a frequency histogram with appropriate binning (Shimazaki and Shinomoto, 2007; Omi and Shinomoto, 2011). For example, in **(C)**, two cases of control and iASD frequency histograms are illustrated. **(D)** *Step 2* is to estimate the parameters of the probability distribution function best characterizing the frequency histograms. For example, in this case, we use the continuous Gamma family of probability distributions to characterize the spectrum of cases from typical to pathological. **(E)** *Step 3* uses the Gamma parameter plane spanned by the shape (*a*) and the scale (*b*) parameters estimated using maximum likelihood estimation (MLE) in this case, with 95% confidence intervals, which we also plot for each estimated pair. **(F)** *Step 4* we obtain new readings every 100 measurements and repeat steps 1–3 to build a stochastic trajectory on the Gamma plane. This trajectory tells us the rate of change of this non-stationary process as the person learns, adapts, or simply performs the same task under different conditions. The stars identify the largest step toward the Gaussian range of the Gamma parameter plane, away from the Exponential range (*a* = *1*) along the shape axis and down toward the regime of lower noise-to-signal levels. Those larger steps can identify the context that will most likely accelerate change in the stochastic signatures toward more symmetric PDFs with lower dispersion.

Specific trajectory parameters of interest included the time series of joint angular velocities (the rate of change of joint orientations over time) and the joint angular acceleration (the rate of change of angular velocity). Figure 1C shows sample trajectories across the body as a child walks. We specifically focused on the peaks of the fluctuations in angular velocity and acceleration. Sample angular velocity peaks are marked in Figure 2A. To avoid allometric effects due to anatomical differences (Lleonart et al., 2000), these peaks are normalized $NmaxV=\frac{maxV}{\left(maxV+\bar{V}\right)}$, where $\bar{V}$ is the average angular velocity of the segment between two valleys (two local speed minima). The minima are automatically obtained from the time series of the speed as a change in slope from negative to positive, while the maxima are detected by a change in slope from positive to negative along the peak angular velocity curve spanned by the various motion types of the participant across the session (Figure 2A). The normalization dictates that rotations that are faster on average, with larger values of $\bar{V}$ in the denominator, produce smaller *NmaxV* values. Lower values of this normalized parameter thus indicate faster angular speed on average. Likewise joint rotations that are on average slower will produce higher values of the normalized peak angular velocity. Figure 2B zooms in one of the peaks. The same normalization procedure was performed on the angular acceleration time series. Since participants ranged between 6 and 19 years of age, all data from all 30 participants were subject to exactly the same normalization procedure in order to compensate for disparities in anatomical sizes. Such disparities impact the overall movement speed (acceleration) and add allometric-size related statistical effects (Mosimann, 1970; Lleonart et al., 2000), thus requiring the normalizing step.

### Second layer of data

The parameters of interest are gathered into a frequency histogram. Here we chose the normalized peak angular velocities and peak angular accelerations to extract the micro-movements waveform. Unimodality of the resulting histogram of micro-movements' peaks is assessed using the Hartigan's dip test (Hartigan and Hartigan, 1985). In the case of failing the unimodality test, the *p*-value is *p* < 0.01. Maximum likelihood estimation (MLE) is used to estimate the shape and the scale parameters of the continuous Gamma family of probability distributions with 95% confidence intervals for the motions of each joint. The empirically estimated stochastic signatures of the whole body motions can then be examined as they distribute by individual body segments or as they integrate across the body (e.g., upper body vs. lower body, specific limbs, etc.), to unveil patterns of the peripheral network of rotational joints.

Building on recent work that empirically parameterized human movements across the spectrum of ages for normal and pathological states (Torres et al., 2016), the continuous Gamma family of probability distributions are used for the distributional analyses and estimation procedures. Previously, linear velocity was shown to be a kinematic parameter that revealed features of various populations in relation to cases of known etiology (Torres et al., 2013a, 2014). Here we focus on the joint angle rotations and their rates of change underlying the displacements of the various body joints. This layer of kinematics provides new insights into the statistics of parameters in the internal configuration space of human rotational joint motions.

The estimated values defining the probability distribution are then plotted for each individual in the Gamma parameter plane, in which typical controls are unambiguously different from pathological cases Figure 2C). Figure 2D shows samples of Gamma probability density functions (PDFs) estimated from the shape and scale parameters across various ranges of kinematics data. We plot in Figure 2E each point (the empirically estimated shape and scale values) from the frequency distributions in Figure 2C to localize the person with 95% confidence intervals, also plotted for each dimension. Note here that each point is a PDF indicating two different probability distributions, a feature that prevents the use of an ideal single theoretical distribution to assess the statistical significance of differences (as is traditionally done). Figure 2F shows that these estimated parameters are non-stationary—rather they shift as a function of context and other extraneous parameters (Torres, 2013; Torres et al., 2013d). In this sense they span a family of probability distributions for each person. The rate of change of these PDFs with context, treatment or mere neurodevelopment of a coping system are possible to evaluate when using these new methods. Utilizing one single PDF theoretically assumed for the population at large is inappropriate when considering kinematics data in general, but in particular it is inappropriate in ASD, a heterogeneous disorder by definition, whereby each person has a unique developmental trajectory.

Using the non-stationary feature of these signatures as the person moves in a naturalistic way, we can obtain new estimates for different conditions within a session (e.g., every N measurements, where N depends on the sampling resolution of the sensors) and quantify the magnitude of the shift on the Gamma parameter plane, i.e., the changes in the PDF that arise as a function of context, treatment, etc. These shifts build a stochastic trajectory that is unique to each person under the conditions of choice. These statistical rates of change constitute yet another layer of analyses that provides useful information about the evolution of the statistical patterns of the person and their unique *individualized* rates of change across the body.

### Third layer of data (indexes of statistical performance)

Along the shape and scale axes of the Gamma parameter plane, we have previously obtained measures linked to the levels of predictability and reliability (respectively) of the motor output signals (Torres, 2013). These levels were previously empirically estimated using stochastic rules to model anticipatory behavior (Torres, 2013) so as to provide a range of values for each individual along the shape axis spanning from random regimes (*a* = *1*, the memoryless exponential distribution) to values of *a* > *10* ranging from skewed to symmetric distributions. In this previous work, shifts to the right of the Gamma parameter plane move the patterns away from randomness (Ross, 2009), toward the Gaussian range. These changes denoted higher certainty in the prediction of impending speeds from past speed and acceleration, taken from trial to trial (Torres, 2013). These rules have served to characterize the statistical signatures of expert athletes with exquisite control and timing of the body movements (Torres, 2011, 2013). The Gamma parameter plane thus serves here to localize each person's signatures of motor performance.

To that end, the moments of the Gamma PDFs were empirically obtained from the estimated shape (*a*) and estimated scale (*b*) parameters whereby the mean $\hat{\mu }=a\cdot b$ and variance $\hat{\sigma }=a\cdot b^{2}$. The noise-to-signal ratio, the Fano Factor (FF) (Fano, 1947), was also obtained from the Gamma statistics to provide information on the stochastic signatures of the normalized peak angular velocity and the peak angular acceleration as a function of other elements (e.g., sex, age, etc.) Substituting the estimated Gamma statistics in $FF=\frac{\hat{\sigma }}{\hat{\mu }}$ shows that *FF* is the *empirically estimated* scale parameter *b*. Therefore, along the scale axis, higher values of *b* denote higher noise-to-signal ratio, and lower values of *b* denote shifts toward more reliable regimes of the continuous random process under study. It should be noted that the term “noise” here has a very precise statistical meaning, in contrast to the negative connotation implied in the motor control literature (Faisal et al., 2008). Noise-to-signal transitions are important markers of adaptive learning. As such, the frequency and amplitude of these transitions on the Gamma parameter plane (phase transitions) are of interest in this framework. In this sense, noise is adaptive when it serves transitions from spontaneous and random to well-structured and systematic or periodic states, but it is detrimental when it stagnates at the spontaneous random levels detectable through our analyses.

### Fourth layer of data (peripheral network visualization)

**Figure 3:**
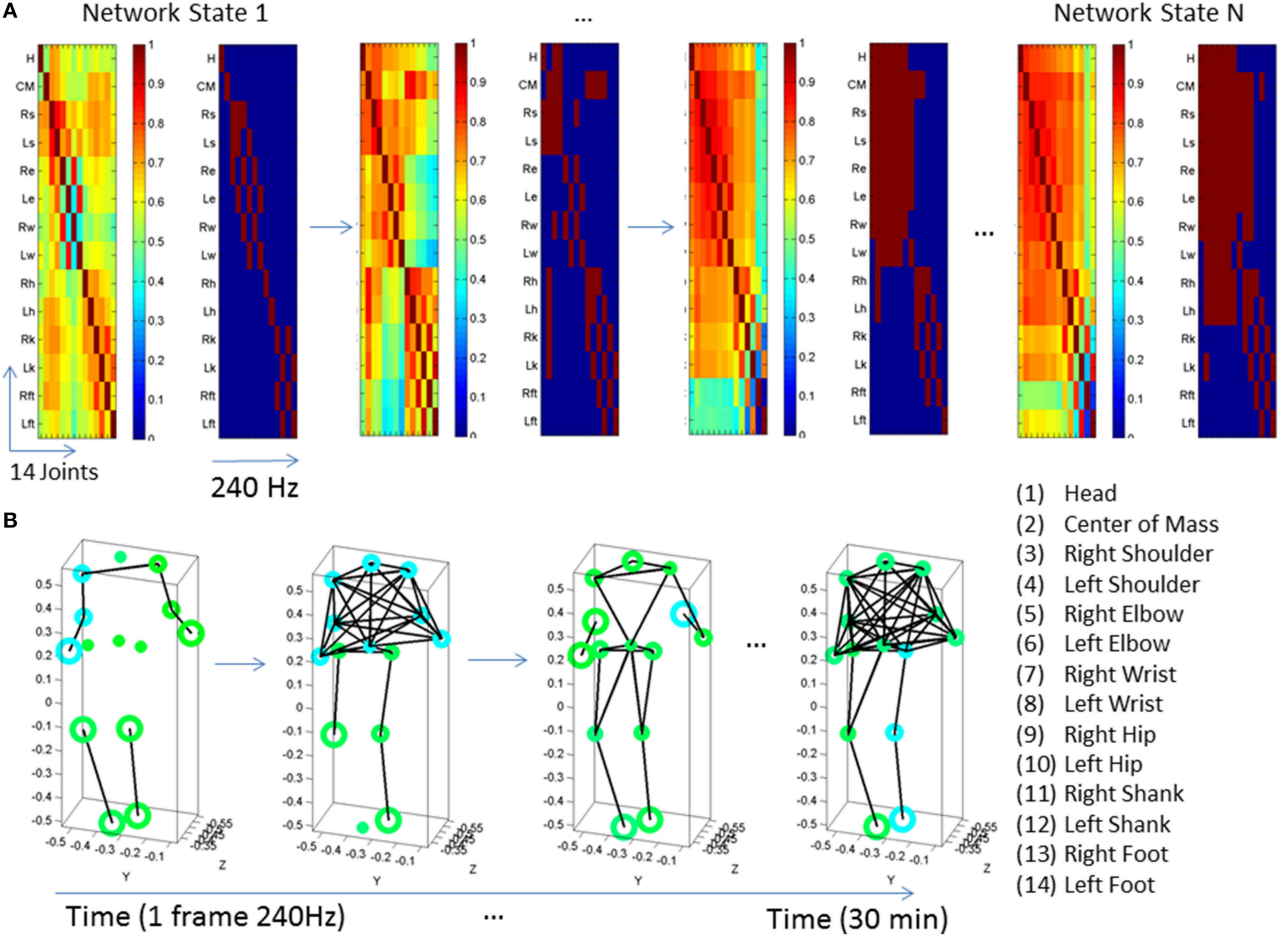
**Visualization of dynamic states of the peripheral network connectivity across 14 joints of the body. (A)** PLV and binary matrices (at 0.85 threshold) showing individual frames from a representative PMS participant. **(B)** Network representation of peripheral joints (nodes) and links (edges) between highly synchronized joints. Circles represent joints and lines are active links within a frame corresponding to highly synchronized joints within a ½h period taken in frames of 240 Hz each. The size of the circle represents a measure of node neighbor-clustering (see text for details). The color of the circle is based on the modularity metric (see text for details). Circles with the same color represent modules of nodes that maximize the number of within-group edges, and minimize the number of between-group edges, i.e., a modular community structure within the network.

Across the joints of the body, we examine the time series of joint angular velocities and obtain the phase locking values (PLV). PLV is a statistic used to quantify the phase coupling between two biological nonlinear signals in time-series, such as time-series of electroencephalographic signals (Gentili et al., 2009; Aydore et al., 2013). In the present study, PLV was employed to quantify the level of coupling (phase synchrony) in the time series of angular velocity values between each one of the 14 joints and all the others. Specifically, the PLV nears 1 in cases where the instantaneous phases of the two joints' angular velocities time series are synchronized. Conversely, if they are unsynchronized the PLV tends to 0. Greater detail regarding the procedure for computing PLV can be found elsewhere [9, 28]. Here we obtained the *14 joints x 14 joints* PLV matrix every 240 frames (240Hz sampling resolution of the sensors). We used a high threshold of synchronization value (0.85) to create a binary matrix. Entries in the original PLV frame that were above or equal to the threshold were set to 1 and those below the threshold were set to 0 (see Figure 3A).

Once we gather the network dynamically evolving frame by frame, we apply tools from the brain connectivity toolbox (Sporns, 2011, 2012) to visualize the temporal profiles of emerging modules and connectivity patterns across this peripheral network of rotational joints. Below are some indexes that we use in these plots.

The ***connectivity index*** is given by the degree of each node (joint), that is, the number of links connecting the node to other nodes in the network. In this case (an undirected, unweighted binary graph), we provide a simplified representation to illustrate densely vs. sparsely interconnected nodes across the network so as to identify critical differences between controls and PMS-ASD cases.

**Figure 4:**
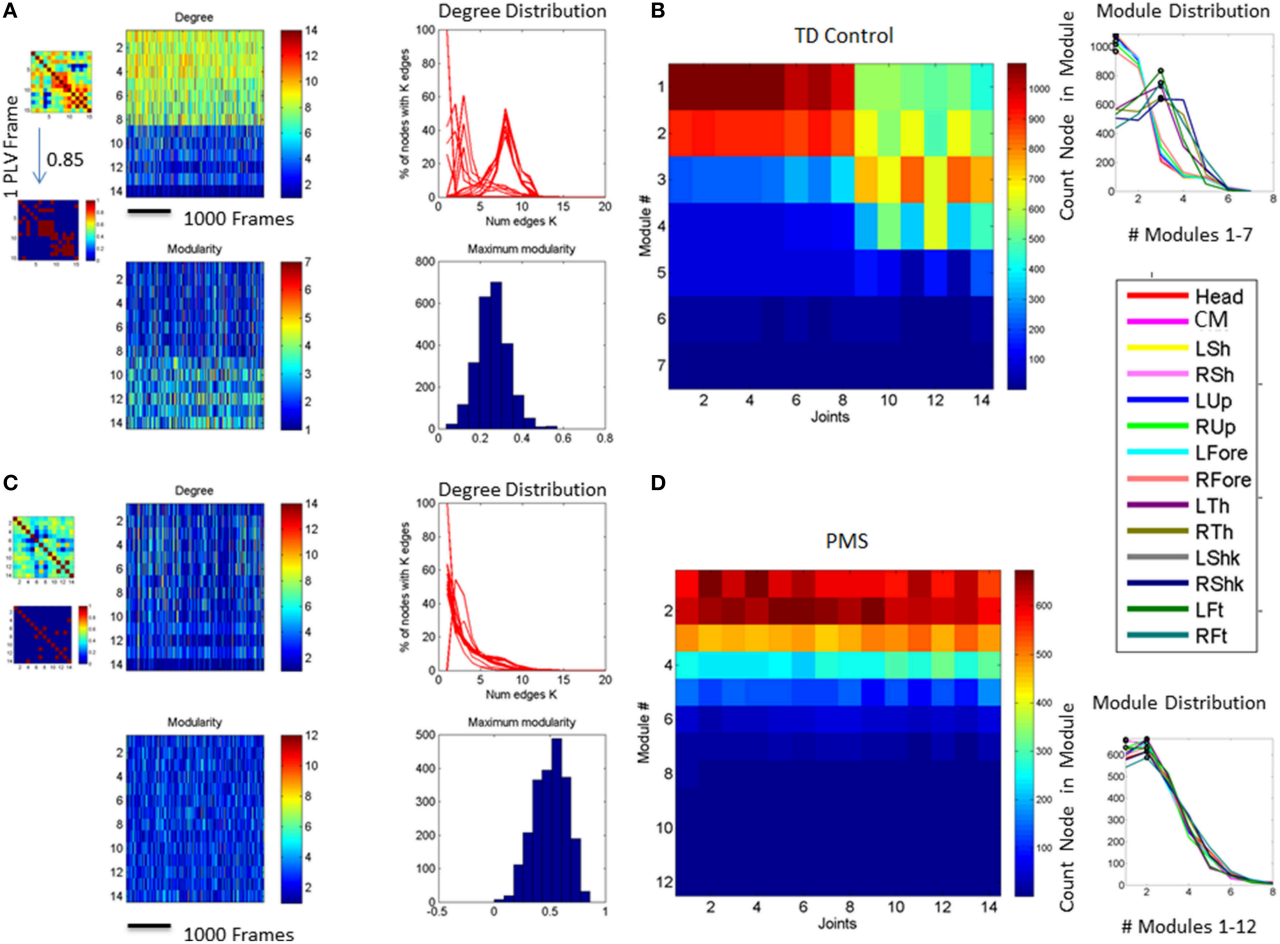
**Network of 14 joints dynamically unfolding in time with self-emerging synergies. (A)** Connectivity matrix 14 joints × 16 s (4000 frames at 240 Hz) from representative control. One frame of the full and the corresponding binary PLV (determined at a 0.85 threshold) are shown as insets. Color bar represents the connectivity. The network has 14 nodes (the 14 joints in the legend of Figure 3 where the center of mass is estimated from thorax and lumbar sensors located as 2 and 3 in the trunk of the avatar in Figure 1) for two representative subjects, one from the typical control group **(A,B)** and one from the PMS group **(C,D)**. The degree distributions of the network and the modularity distributions are obtained from a 30 min walk registering fluctuations at 240 Hz. Notice the contrast between the two representative participants. **(A)** also shows on the left the full and binary PLV matrices of one frame at a 0.85 threshold (high synchronicity). In the 14 joints × time (frames) matrix each entry provides the degree for each node (joint) as reflected in the color bar. There is higher connectivity in the upper body (numbers 1–14 are as in legend of Figure 3) than in the lower body for this typical participant. The degree distribution of the network for a segment of the 30 min walk is also shown on the right-top panel. Notice that the curves represent the degree distribution of the joints and that a pattern emerges corresponding to the upper and lower body as well. Specifically the upper body has distributions centered farther to the right, with higher number of links than the lower body. The lower body has more nodes with fewer links and fewer “hub” nodes (in the tail of the distribution). Notice that the upper body has higher connectivity than the lower body in this participant (joints are as in the legend of Figure 3). Degree distribution identifies two distinct groups of nodes. Corresponding modularity matrix identifies up to 7 modules (color bar). Frequency histogram of maximal modularity is also shown. **(B)** Matrix quantifying the joint participation per module (color bar shows the counts). Inset shows the module distribution with identification of modules with maximal count per joint participation. Two modules self-emerge (1 and 3) as the ones with maximal joint participation denoting two main synergies summarizing the complex 14 joint patterns of the gait in this participant. Colors in the legend identify the joints participating in the two synergies. Notice that one contains the upper body joints and the other the lower-body joints, consistent with the degree distribution in **(A)**. **(C,D)** The same information as in **(A,B)** is shown for a PMS participant. Notice the striking differences with the control and the lack of synergies in the PMS case.

The ***modularity index*** provides a sense of the subdivision of the network into non-overlapping groups of nodes that work together within a “community.” This metric assesses self-emerging structuring of the network by maximizing the number of within-group edges, and minimizing the number of between-group edges. Thus, modularity is a statistic that quantifies the degree to which the network may be subdivided into such clearly delineated groups. For example, Figure 3B shows the contrast in modularity (green vs. cyan node colors) as the connectivity differs between the upper and lower body and changes frame by frame. This is better appreciated in Figure 4 unfolding the walking session over time, frame by frame within the 30 min walk period, as well as helping us identify synergies (see explanation on synergies below).

The ***clustering coefficient*** another metric that we can use is the clustering coefficient, i.e., the fraction of triangles around a node, or equivalently, the fraction of node's neighbors that are neighbors of each other. For example, in Figure 3B the size of the circles at each node is given by the clustering coefficient value. Large circles indicate nodes whose neighbors are neighbors to each other. Figure 3B shows snapshots of the evolution of the network for different frames. In summary, lines represent links between nodes. Circles at the node track modularity (color coded) and clustering coefficient values (size of the circle).

This is an arbitrary choice of metrics for visualization and analytical purposes. They are tools that we have adapted (for the first time) from the analyses of central networks of brain nodes (e.g., from electroencephalography) to the peripheral network of joints from bodily rhythms. These modified methods help examine the self-structuring of highly complex motions spanned by the many degrees of freedom (DoF) of the body. They complement our previous work on detection of synergies and co-articulation in goal directed behavior (Torres and Zipser, 2002, 2004; Torres and Andersen, 2006; Torres et al., 2011) and provide new tools for examination of highly automated behaviors such as walking.

For more formal explanations on the mathematical foundations of network analyses we invite the reader to consult various networks books (Newman, 2003; Newman et al., 2006) and network references with more traditional applications to brain networks (Sporns, 2011). Notice here that the peripheral sensory and motor nerves which we “listen” to with the sensors are an extension of the brain networks. The motions they register are controlled by the brain both under volition, as well as spontaneously (Torres, 2011; Torres et al., 2011). Thus the extension of network analyses from the brain nodes to the peripheral nodes is not only justified, it is actually strikingly informative and simplifying of an otherwise intractable DoF redundancy problem (Bernstein, 1967; Torres and Zipser, 2002).

### Fifth layer of data: identification of synergies in the network

The unfolding information on connectivity/modularity could be important in tracking efficient distribution of activity across the network and proper recruitment of the body's DoF to boost automatic coordination across body parts (e.g., between upper- and lower-body). By examining synchronous sub-networks we can uncover self-emerging synergies and co-articulation patterns at the joint angles level that may inform individual self-regulatory motor control strategies. The motivation here is that examination of a coping neurobiological system -such as that of PMS children- may reveal self-emerging / self-discovered over-compensatory strategies by the nervous systems of some of the children that could help us guide treatment in others with iASD. More importantly the personalized treatment of the problem can help us tailor interventions involving sensory substitution and sensory augmentation therapies well suited to the unique bodily characteristics of each child as the brain tries to control its peripheral joint network dynamics.

We refer the reader to Figure 4 for further details on the connectivity metrics illustrated by contrasting two representative subjects from the control and the PMS groups.

In Figure 4B the modularity index is tracked for each frame and represented in matrix form as well (14 joints × time). Each entry contains the module number where the joint participated in a given frame. The color bar indicates the maximal number of modules emerging in the network across the 30 min walk. In this case up to 7 modules self-emerge throughout the session. The modularity distribution is also obtained by counting across the session the participation of each node (joint) in each module. The joints color coded are identified in the legend. These distributions reveal distinct self-emerging synergies of the joints in the upper body and the lower body. The module with the maximum count of joint participation is obtained for each joint. The joints are gathered per module so as to define the synergies (the set of joints maximally participating in a given module). Take for instance the Figure 4B. The first two modules comprise joints from the upper (1–7) and lower body (8–14), but those of the upper body define a synergy because they have maximal count. Thus the first two modules identify a synergy with maximal joint participation from the upper-body. The third module defines another synergy as per maximal participation of joints from the lower extremities. Clearly, walking patterns are not as complex as those of a sports routine or a ballet routine (for example). In the latter more synergies would typically emerge across the performance. Yet in all cases the self-emerging synergies can be automatically detected using the present biometrics to define a set of articulated motions with the potential to reduce the complexity of the body's DoF to a well-defined vocabulary of actions.

Notice the marked contrast captured by these personalized metrics between the representative control and the representative PMS participant who shows very low connectivity, and very homogeneous modularity distributions. This outcome suggests that the body of the child with PMS does not coordinate synergies as the typical control does. Instead a larger number of independent modules emerge with homogeneous distributions of joint participation across all modules and no synergistic patterns efficiently co-articulating the joints. This is a sign of very atypical motor control requiring far more physical energy to manage many independent modules than to manage a few synergies.

### Roadmap and motivation for the order of analyses

We examined the statistical patterns of fluctuations in motor performance (micro-movements) across the body. First we characterized the normative patterns of angular velocity in the feet and identified fundamental statistical differences in the PMS and iASD children with respect to the normative data from controls of various age groups. We then examined the underlying patterns of angular acceleration of the lower body, specifically the legs, and characterized the noise levels of their micro-motions. We then moved up to the upper limbs and examined patterns of the arms' micro-motions during their overall body movements when walking back and forth in the platform of Figure 1. This analysis revealed statistical differences (different probability distributions) between typical males and females that were even greater in the PMS children, suggesting a possible way to differentiate the PMS male and female phenotype. Lastly, we examined synchronization patterns of angular velocity across the body and uncovered striking differences in the network's connectivity metrics. Despite their heterogeneity, the connectivity patterns were unique across the three iASD individuals. The patterns of modularity revealed differential synergistic and coordination patterns between the upper and the lower body extremities in PMS and iASD in relation to controls.

## Results

### Atypical foot-turning angular velocity patterns in PMS-ASD

**Figure 5:**
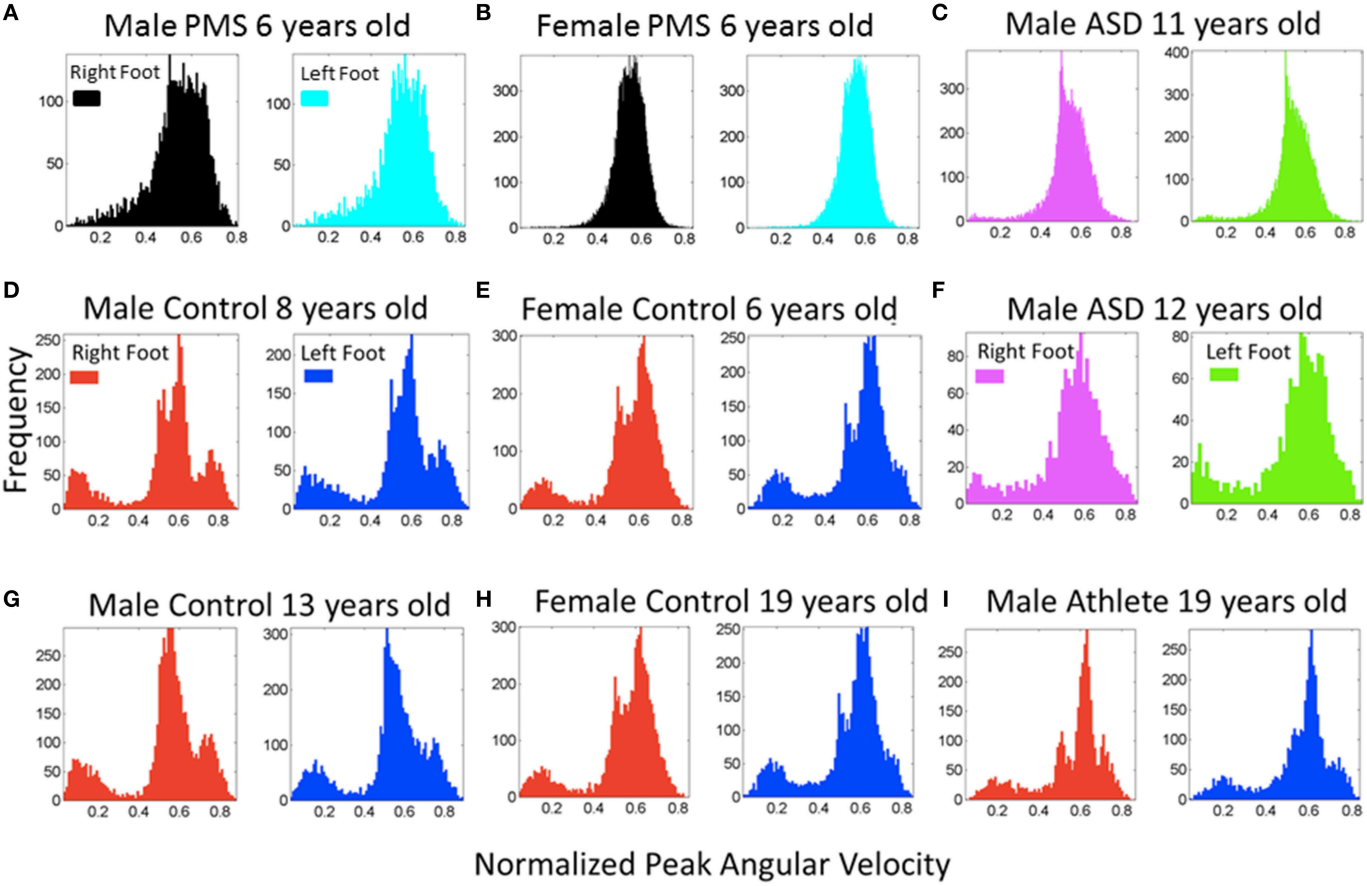
**Frequency histograms of the normalized peak angular velocity for the footsteps**. **(A,B)** Representative patterns from PMS participants (6 years old) show a unimodal distribution of the normalized joint angular velocity index. **(D,E,G,H,I)** Representative controls of a broad range of ages including ages and sex of the PMS participants show multimodal distributions with a systematic bump of smaller values of this index indicating a density of faster rotations on average (the denominator of the index containing the average angular velocity between the two local minima is higher). **(C,F)** Participants with idiopathic autism spectrum show both a lack of faster rotations in the turns **(C)** quantified in the PMS and the presence of them **(F)** quantified in controls. **(I)** This panel shows the participant with ideal patterns, a martial arts expert with fine control, timing, and joint coordination in his movements.

Examination of the frequency histograms of the normalized peak angular velocity of the feet revealed that children in the typical control group all failed the Hartigan's dip unimodality test (*p* < 0.01) as all children manifested multimodal distributions (Supplementary Figures 1, 2). Representative control children of 6, 8, and 13 years old (the ages of PMS children) are displayed in Figures 5D,E,G. Representative controls of college age are shown in Figures 5H,I. This result indicates that the walking of typically developing children produced distributions with multiple “bumps.” The densest bump accounted for slower rotations, while the smaller bumps could contain slower or faster rotations relative to the largest (densest) bump. In stark contrast, the children in the PMS group did not show the faster rotations' bump. Closer inspection of the peaks in the multiple bumps revealed that several of the faster rotations that were absent from the PMS but present in the controls, occurred during the turns, while the other rotations in the densest bump were part of the walking patterns.

Figure 5 also shows sample frequency histograms from representative subjects in the PMS group, one male (Figure 5A) and one female (Figure 5B) PMS contrasting with the Figures 5D,E control children of same or comparable ages.Figures 5C,F depict two of the children with iASD. Not surprisingly, the iASD features for this parameter were heterogeneous (2/3 had similar unimodal distribution patterns as those of the PMS but we will see later that overall body patterns are unique in these three very heterogeneous children). Figure 5I shows a teen control who is also a martial arts expert. The martial arts expert is a black belt second-degree karate expert who has competed and trained since approximately 4 years of age. This subject's data provides a reference for the ideal case scenario of fine motor control and head-trunk-limbs coordination.

**Figure 6:**
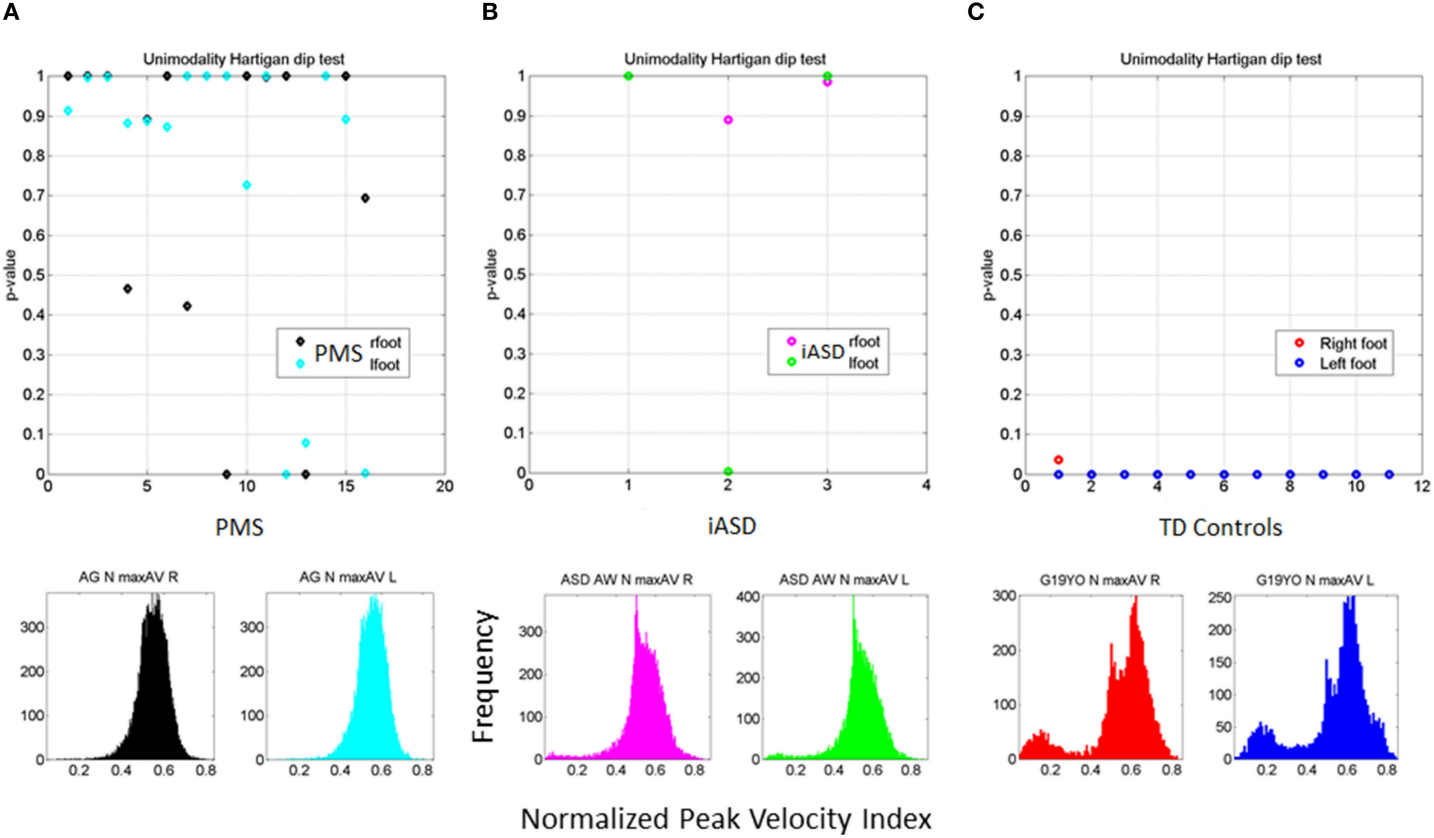
**Outcome of the Hartigan's unimodality dip test. (A)** The *p*-values > 0.05 from the Hartigan's dip test of unimodality from the distributions of the normalized peak angular velocity index in 16 PMS children ranging between 5 and 16 years old show systematic lack of multimodal distributions in one or both feet. **(B)** Patterns of iASD children show consistent *p* > 0.05 patterns for one or both feet. Representative histograms for right and left foot are also shown. **(C)** Patterns from control participants (ages 5–19 years old) had *p* < 0.01 thus failing the unimodality dip test for both feet. Representative histograms for both feet of one participant are shown.

All PMS children display unimodal patterns (non-significantly multimodal patterns) according to the Hartigan's dip test of unimodality, (*p* > 0.1) for one foot or for both feet (see Figure 6A). There is an asymmetry in the results of some of the PMS children whereby one foot reaches *p*-values close to significance (the signature of multimodality found in controls) but the other does not. In subsequent analysis of whole body connectivity patterns, it is evident that these are children whose upper body joint network patterns approach those of typical controls. Figure 6B shows results from the 3 children with iASD. Figure 6C shows the results from the controls. The PMS patterns (Hartigan's dip test of unimodality *p* > 0.1) stand in marked contrast with the neurotypical controls (Hartigan's dip test of unimodality *p* < 0.01).

### Legs patterns are random and noisy in PMS children

**Figure 7:**
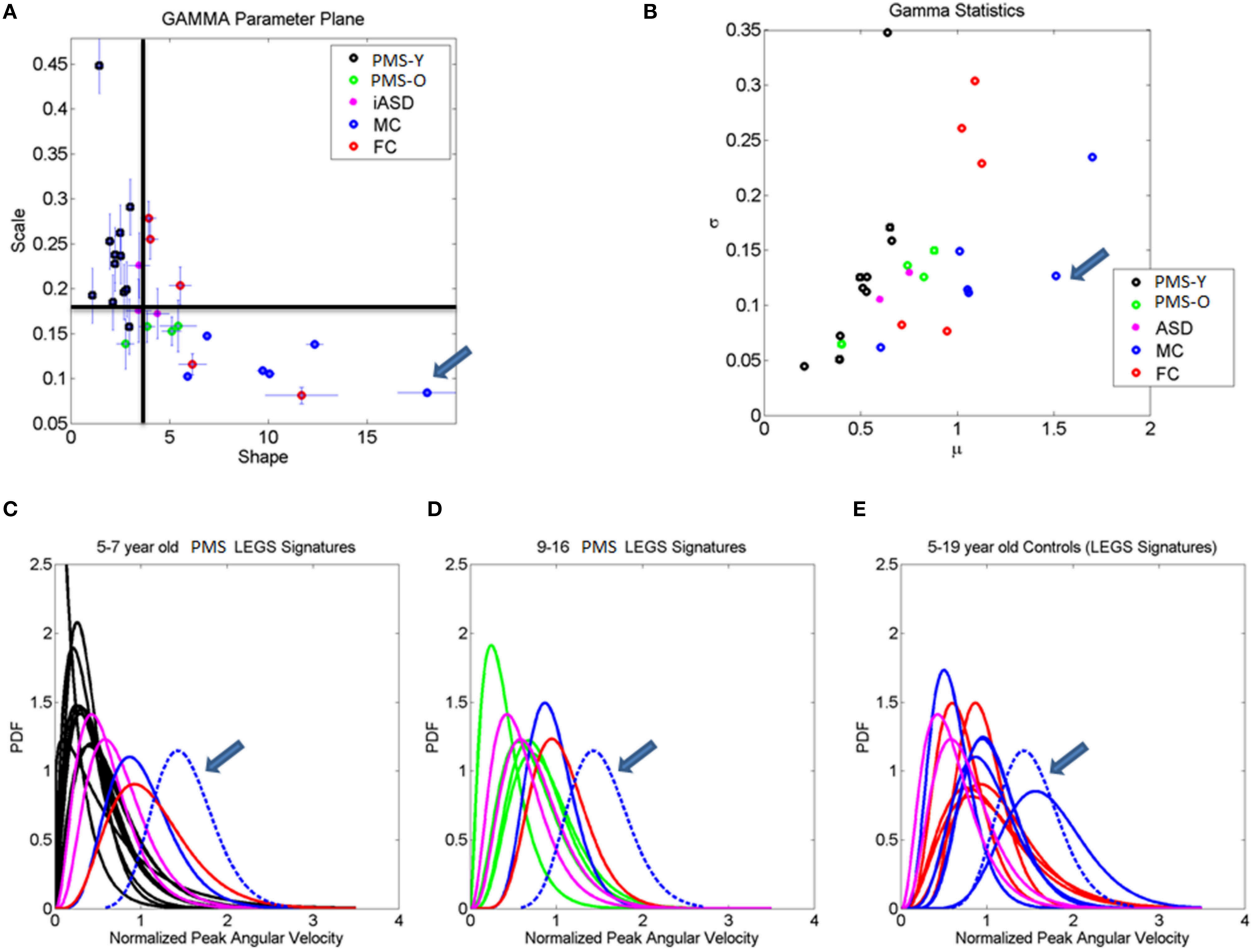
**Stochastic signatures of micro-movements in peak angular acceleration from the legs during gait**. **(A)** The empirically estimated shape and scale Gamma parameters from the normalized peak angular velocity index of the legs are plotted for each child with 95% confidence intervals. The lines denote the median shape and scale values automatically dividing the Gamma parameter plane into four quadrants. All but one younger PMS children (PMS-Y) 5–7 years old fall in the left-upward quadrant with highest noise and shape values close to 1 (especial case of the Exponential distribution). The farthest to the right (symmetric distribution) and lowest scale value (lowest noise to signal ratio) is a professional male athlete (19 years old) marked by the arrow. Green dots are older 9, 12, 14, and 16 year old PMS (PMS-O). **(B)** Empirically estimated Gamma mean and variance with similar color scheme as **(A)**. Higher values of the mean normalized peak velocity index indicate slower angular velocity on average. **(C–E)** Empirically estimated Gamma PDFs for each participant using the same color code scheme as in **(A,B)** according to age for the PMS and sex for the controls. ASD participants are plotted as reference. In each panel **(C,D)** with the PDFs from PMS children, we plot the PDFs of age- and sex-matched controls. Red (FC) and blue (MC) are typical female and male controls respectively. **(E)** All controls and ASD participants are plotted and the PDF of the professional athlete stands out as the Gaussian shape with lowest dispersion in the cohort [see his shape and scale parameter range in **(A)**].

Building on the results from the foot patterns, the patterns of variability in the micro-movements of the legs' angular velocity were assessed individually (Figure 7). The results for each child are plotted on the Gamma parameter plane with 95% confidence intervals for each estimated shape and scale dimensions. The median of each Gamma parameter across the cohort was obtained to identify the different quadrants of the scatter (as divided by the two lines). Note that values in the upper left quadrant of the noise-to-signal ratio (the Fano Factor which is also the scale parameter) are higher and the values of the shape parameter are closer to *a = 1*, the memoryless Exponential distribution (Figure 7A). All children with PMS between 5 and 9 years old fell on or close to this noisy and random quadrant, as well as 2/3 of the children with iASD (those with the unimodal distribution of feet's normalized peak angular velocity index similar to the PMS). This localization of the micro-movements signatures suggests leg patterns that are harder to predict from moment to moment, as they have higher noise and higher uncertainty than those in the lowest-rightmost quadrant where the older controls with mature gait patterns fell.

The lowest-rightmost quadrant contains all the controls above 13 years old and three of the PMS adolescents (Figure 7A). The lowest-rightmost quadrant is also the region of the lowest noise-to-signal ratio. At the extreme lowest-right most corner of the quadrant (marked by an arrow) is the athlete with the ideal statistical patterns of micro-motions, with a Gaussian-like probability distribution function, and the lowest levels of noise.

The three control females that fell on the boundary (median shape value and above the median scale value), with higher noise to signal ratio than other controls are 5–6 years old. At this age the gait patterns are still maturing in the typically developing children (Sutherland et al., 1980; Berger et al., 1984; Stolze et al., 1997). Not surprisingly, the stochastic signatures of walking patterns in these younger children are not yet localized with the older controls above 8 years of age. The latter have developed a mature gait pattern, so their overall walking patterns have statistically more controllable and predictable features.

Figure 7B shows the estimated Gamma statistics corresponding to the estimated Gamma parameters in Figure 7A. The three young control females have high variability, yet their joints rotate slower than those of the PMS children. Their higher mean values of the normalized peak angular velocity index indicate that the average rates of joint rotations (in the denominator of this index) is lower, which provides more capacity to control the joints. In contrast, the PMS children with lower mean values indicate faster rates of joint rotations, a feature that may impede controllability of the legs. Figures 7C,D show the empirically estimated probability density functions (PDF's) for the children with PMS. Panel C contains all the children who did not fall in the lower-right quadrant delineated by the median values of the Gamma parameters. These happened to be the younger group (5–7 years old). We also plot in this graph the PDF's of the neurotypical control children of comparable ages and the ideal athlete with Gaussian PDF, highlighted with an arrow. Notice that these PDF's are the most skewed of the group, toward the exponential shape. The iASD patterns are also plotted for reference. In addition to the skewed shapes they have the highest dispersion.

The older PMS (9–16 years old) in Figure 7D fell in the rightmost lower quadrant. They have a notable shift in patterns with respect to the younger PMS. Notice as well that the iASD are all 11–12 years old and also differ from controls in that age range. Figure 7E shows the broad distribution ranges of PDF's across the typical controls 5–19 years of age for both sexes, with the ideal pattern of the athlete highlighted as well. This figure underscores the striking differences in statistical patterns across typical development as well as their evolution from distributions with highly skewed shapes to those tending to the Gaussian shape, accompanied by a decrease with age in the dispersion (noise to signal). It also shows the range of values of the parameter of interest, the normalized peak angular velocity index. Younger children have a faster rate of rotation on average (smaller values of the index) than older controls. This indicates that on average the joints of the older participants rotate at a slower rate, possibly enabling better controllability.

### Lower and upper body connectivity patterns differ between PMS, IASD, and controls

Finally, we examined connectivity and modularity patterns of the peripheral network of joints. A controllable system must have a good balance between highly interconnected nodes and independence in self-emerging modules that appropriately exploit the abundant degrees of freedom of the body through efficient synergies and patterns of co-articulation. We tested several indexes related to coordination.

**Figure 8:**
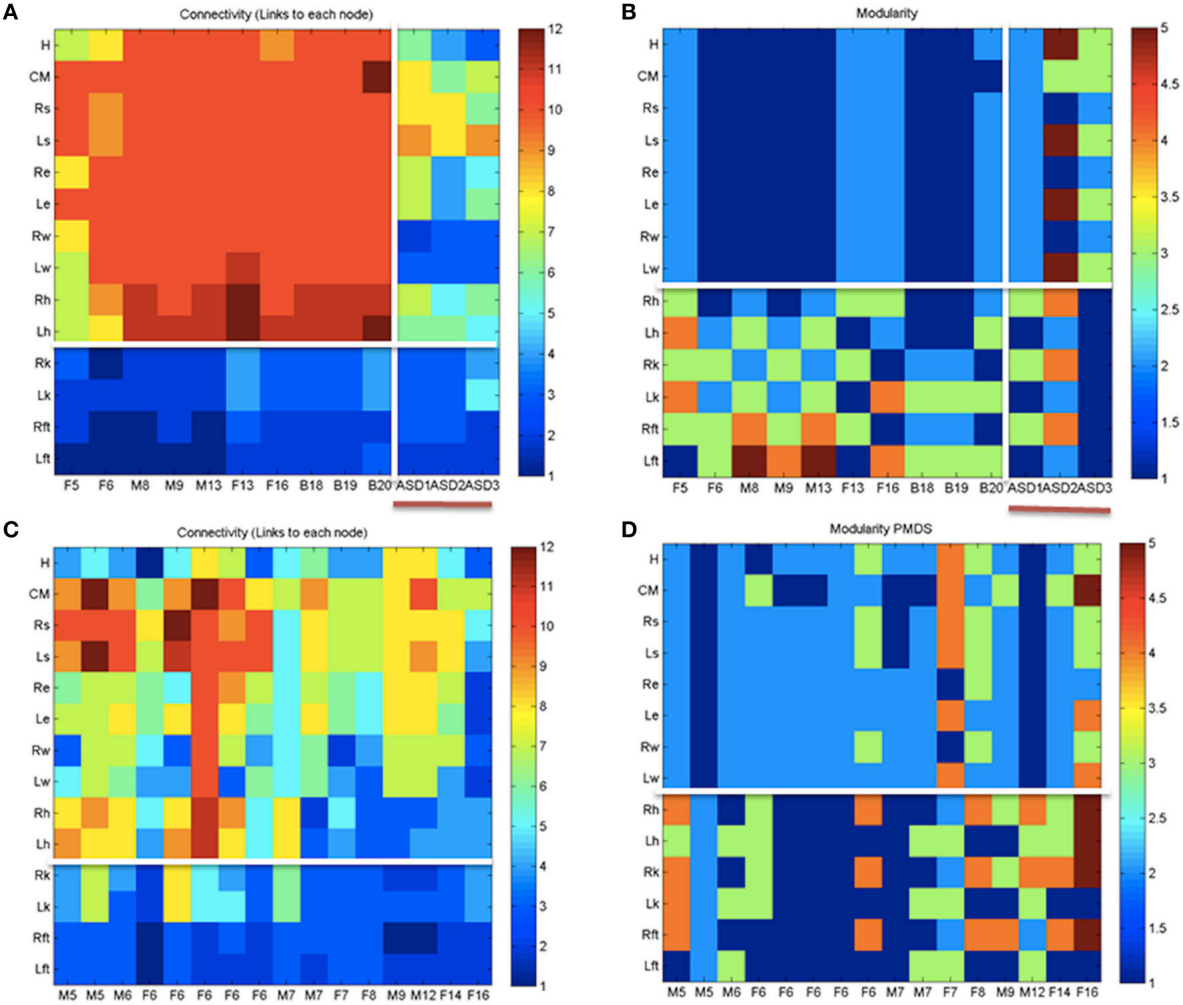
**Indexes of peripheral network connectivity and modularity across subjects**. **(A)** Summary patterns for controls and the 3 iASD participants. Each column represents a participant's pattern of network connectivity from low (blue) to high (red) degree of connectivity. The last three columns separated by the vertical white line are the 3 iASD (9–12 years old with connectivity patterns close to the 5 year old in the first column). Labels in the column axis are sex-age (e.g., F5 means female 5 years old). Each row represents 1 of 14 joints across the body as in Figure 4 legend. Above the white line are head, center of mass, right shoulder, left shoulder, right elbow, left elbow, right wrist, left wrist, right hip, and left hip. Below the white line are right knee, left knee, right ankle, left ankle. **(B)** Average modularity index taken across the 30 min walk for control subjects (last 3 columns are the iASD). Horizontal line divides the upper body from the lower body. **(C)** Summary connectivity matrix for PMS children. **(D)** Summary modularity matrix for the PMS children.

Figure 8 shows the overall connectivity patterns encountered in the typical controls from 5 to 19 years old as well as those from the 3 iASD children. Notice that the patterns of the latter resemble those of the typical representative 5 year old child, even though the iASD children are 10–12 years of age. The controls have a highly interconnected network in the upper body and the hips with certain independence with the legs and feet (Figure 8A). In stark contrast, the patterns of the PMS children are notably different as demonstrated in Figure 8C, with low to none connectivity across the upper body and some interdependences with the legs that are absent in the controls. The modularity indexes also revealed striking differences between controls and PMS. These are shown in Figures 8B,D respectively. From the hips down the controls show modularity of variable degrees that contrast with 2 modules at most in the upper body. These must result in very different coordination patterns in relation to the PMS patterns. It suggests that the recruitment and release of degrees of freedom across the body is highly atypical in PMS. We further explore this point by dynamically unfolding the above averaged patterns across the 30 min walk session.

**Figure 9:**
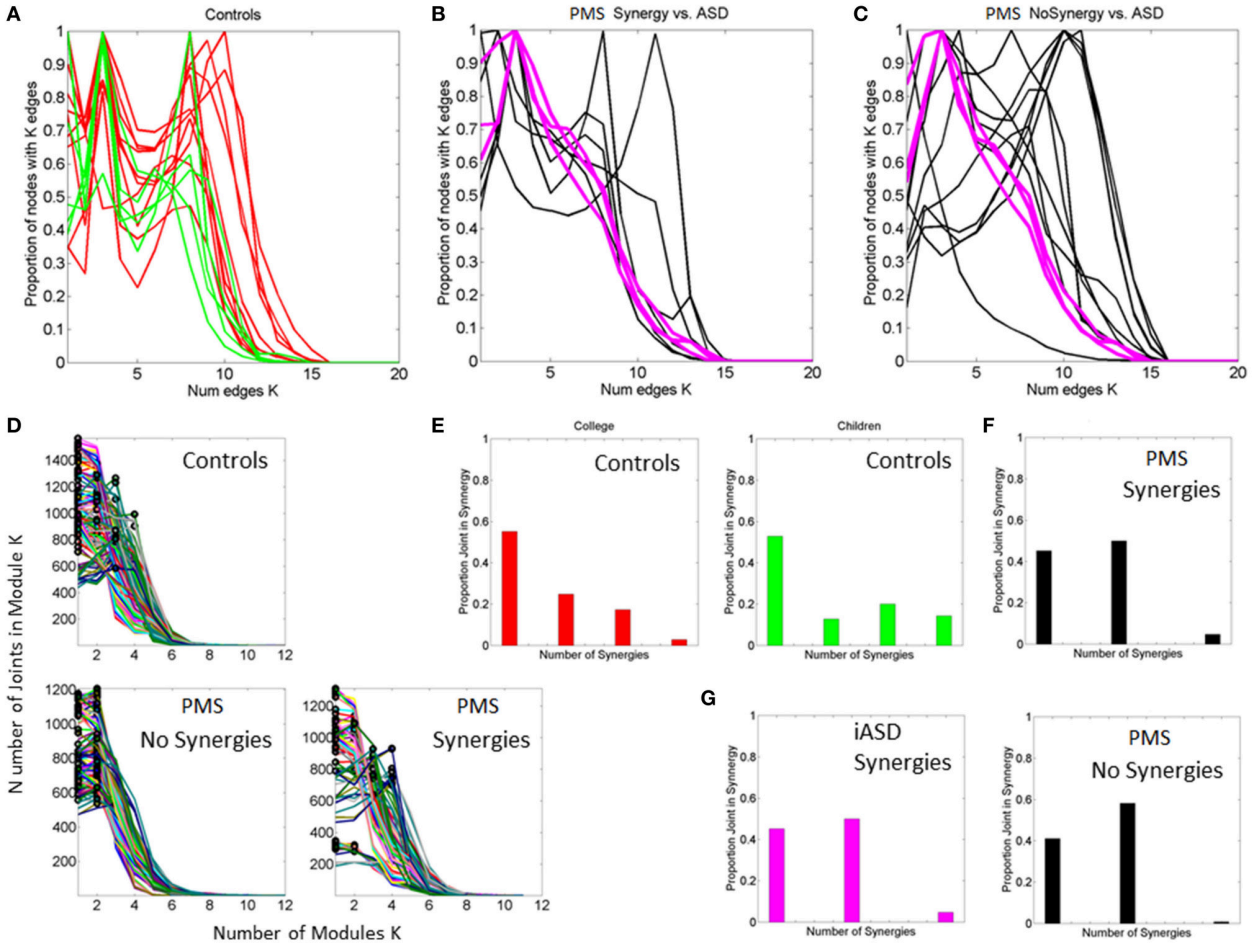
**Connectivity and modularity indexes dynamically obtained from all frames. (A)** Degree distribution over all frames for each control participant (red represent college, green represent children matching the age and sex of PMS). **(B)** Degree distributions of PMS participants with synergies of the upper body. Patterns of iASD are shown for reference (notice their homogeneity in this metric despite their heterogeneous clinical phenotype). **(C)** Degree distributions across all frames from PMS with no synergies superimposed on the three iASD. **(D)** All distributions of node (joint) participation in modules. Black dot represents the modules with maximal node participation (a synergy is a module with maximal participation from multiple joints). PMS (11/16) with no synergies (see also Supplementary Figure 1). In these cases all joints participate homogenously in the module. They are contrasted with PMS with synergies (5/16) recruiting upper body joints but none in the lower body. **(E)** Number of synergies vs. proportion of joints participating in module for college and children controls. **(F)** Same as **(E)** for PMS with upper body synergies. **(G)** iASD with similar patterns as PMS with synergies and PMS with no synergies.

Figure 9 further expands the results on connectivity and modularity across the 30 min walk session. Figure 9A shows the degree distribution for all controls. This figure shows two distinct peaks in the distribution whereby there is a large proportion of nodes with 3 to 4 links and another large proportion of nodes with 7–11 links, suggesting -for each subject- a main hub in the network and also a number of nodes potentially working more independently. By contrast the PMS show more variable connectivity patterns across the cohort divided into those which show a synergy and those who do not. They either have a single peak corresponding to a large proportion of nodes with a high number of links but an absence of nodes with low connectivity, or they may have the opposite pattern. The iASD have similar patterns of connectivity consisting of a high proportion of nodes with low number of edges (Figures 9B,C). This implies that the nodes do not form modules putatively leading to efficient co-articulation and synergies required for good coordination. Instead, for all three iASD children, the joints seem to be working independently with poor coordination. Figure 9D shows the patterns of synergies identified in controls (as per methods Figure 4) and contrast them with those in PMS. The latter broke down into two groups that corresponded as well to the Figures 9B,C with different underlying connectivity patterns. One group has no synergies of the upper or lower body so the center of mass, arms and legs are not well coordinated. The other has some upper body synergy but also an absence of synergies in the lower body. These patterns are also shown in panels Figure 9E for the controls and Figures 9F,G for the PMS and iASD (see also Supplementary Figure 3). The bar plot counting the proportion of joints involved in the synergies show higher count in controls with a break down into 4 synergies across the body. The lower count and different distribution of synergies is evident in the PMS and iASD. In both cases the coordination patterns across bodily joints are bound to be very poor or absent.

### Systemic noise and randomness separate PMS from controls

Notice that in Figure 7E the controls span a broad range of shape values, from left highly skewed to the rightmost Gaussian. In contrast, the ASD and PMS groups have narrower sets of PDF shapes. The PMS group is the closest to the exponential, while the ASD group has the narrowest range. This lack of diversification across the joints (14 × 3) in ASD children with very different clinical phenotypes (one non-verbal extremely violent, one non-verbal visibly hypotonic and one extremely verbal with additional features of Tourette's and ADHD), indicates that despite the heterogeneity of idiopathic-ASD, there are two emerging common features from this study that coincide with previous findings (Torres et al., 2013a): (1) the narrow bandwidth of shapes from the empirically estimated probability distributions of the continuous Gamma family, and (2) the high levels of sensory-motor noise extracted from the micro-movements.

The distributions of the noise-to-signal ratio (the scale parameter) empirically estimated from the angular acceleration across all body joints were also found to differ across subjects (significant differences from non-parametric Friedman Test, *p* < 0.01). The PMS group has mean noise values comparable to mean noise found in controls (due to pooling across the different ages) but has an overall broader range, particularly in the higher-noise end (contributed to by the younger children in the group, multi-compare test upon non-parametric ANOVA Kruskal-Wallis test, with significant differences between young and older PMS, Chi-square, *p* < 0.01).

Supplementary Figures 4A,B show the empirically estimated Gamma statistics (first and second moments) obtained from the estimated shape and scale Gamma parameters. Here the PMS patterns show the lowest mean value in the normalized peak angular acceleration index. This indicates that the underlying average rate of change of angular rotation across the joints is high (denominator term), thus confirming (1) the highly accelerated state of their joint rotations; (2) the possible lack of controllability found in the legs' actual rotational acceleration patterns. This result implies a systemic lack of controllability in PMS that is not present in the joints of the iASD children under study here. These iASD children span mean values comparable to controls, but have higher variance than age and sex-matched controls and the PMS group. To test for differences in variance we used the 3 control male children in the range of 8–13 years old and the 3 iASD males of comparable ages (3 children × 14 joints × 1000 randomly chosen measurements of normalized peak angular acceleration index to estimate the Gamma moments) and tested the differences in estimated variances between controls and iASD in a non-parametric one-way ANOVA Kruskal-Wallis test (*p* < 0.01).

**Figure 10:**
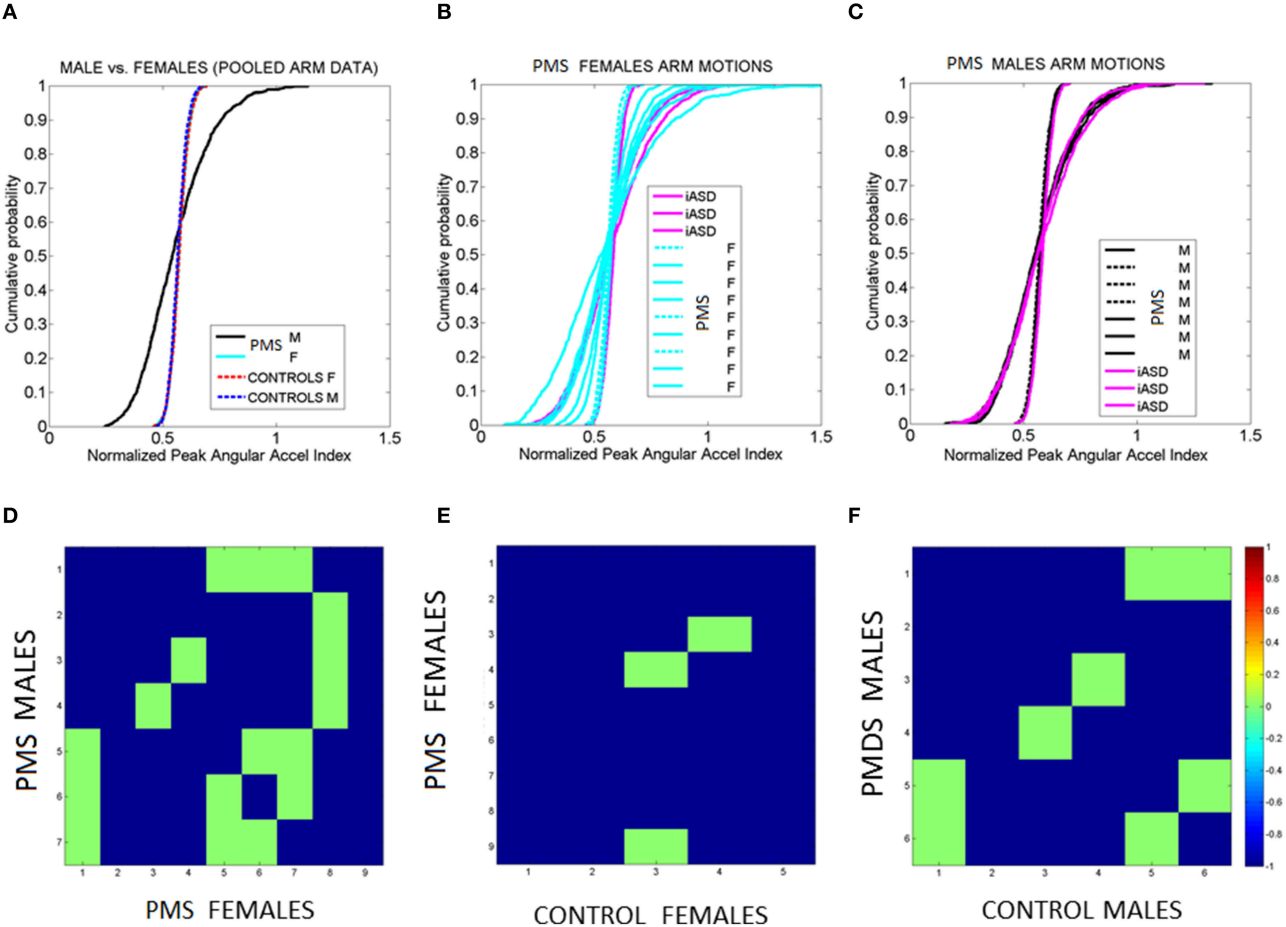
**Fluctuations in arms' angular acceleration distinguish sex in PMS. (A)** Empirical cumulative distribution function (eCDF) estimated from the fluctuations in normalized peak angular acceleration index obtained from arm motions during walking Data are pooled across all males and all females with PMS vs. all male and all female controls. **(B)** eCDF from each PMS female in relation to the three iASD (dashed curves fall near female control signatures). **(C)** eCDF from each PMS male in relation to the three iASD (dashed curves fall near male control signatures). Notice the contrast with **(B)**. **(D–F)** Pairwise comparison matrices drawn using Kolmogorov-Smirnov test of two eCDFs. Entries are the log of the *p*-value from the test. Color bar indicates the log *p*-values. Entries colored in green are 0.05–0.07, thus failing to reject the null hypothesis that the differences in eCDFs are statistically significant at the 0.01 level. Blue entries mark near zero *p*-values, significant at the 0.01 level. Notice that 2 iASD fall with the larger number of PMS males and one iASD falls with the smaller number of PMS males.

While the PMS group suffers from the systemic excess rotational acceleration issue, the iASD group suffers from systemically higher noise levels in their micro-movements. This is consistent with the faster rate of accumulation in the iASD empirically estimated cumulative distribution functions (eCDF), see Figures 10C,D. This extends previous results on hand micro-movements in ASD to full-body, systemic levels (Torres et al., 2013a). Peripheral noise, which is absent in mature controls of comparable age, is indeed present in iASD and in PMS, particularly at the younger 5–7 years of age. Note that the iASD participants are older than 7 years of age (10–12 years of age). A possible interpretation is that excess noise may prevent proper maturation of other aspects of walking; because such processes require statistical estimation and prediction of bodily biomechanical states, a very challenging thing to do under persistent noise and uncertainty.

### Arm patterns differentiate sex in PMS

Building on the differences in upper- and lower- body synergies between controls and children with PMS and iASD, we investigated variations in upper body extremities during the walking. To this end we empirically estimated the Gamma parameters from the data of each of the female and male subjects and generated 1000 points per group by randomly choosing from the empirical parameter values of the normalized peak acceleration index.

Figures 10A–C shows the results of examining the normalized peak angular acceleration index. In Figure 10A the data is pooled across all males and all females PMS and the eCDF is obtained. The results of the corresponding pairwise individualized comparisons using the Kolmogorov-Smirnov test for two eCDFs are shown in matrix form in Figure 10D. Each entry represents the log of the *p*-value color coded according to significance level (see color bar). Notice that all *p*-values are extremely small. Even the non-significant entries are very close to 0.05. The most striking feature of the pooled data is the clear separation between males and females in PMS. The differences are also appreciated in the individual eCDFs shown in Figures 10B,C for females and males with PMS respectively, with corresponding *p*-value matrices shown in Figures 10E,F respectively. In each plot the three iASD were also plotted for reference. Notice that two iASD align with the PMS males and one iASD aligns within typical ranges. As a group, the fluctuations in arm motions in PMS children during walking unambiguously distinguish males from females. When unfolding the data individually the females with PMS show a broader range of variability in eCDF patterns than the males. Further comparison between PMS females and control females (or PMS males and control males) show differences in the empirically estimated distributions as well. Such differences are not captured by criteria from DSM or ADOS-G (Autism Diagnostic Observation Schedule—Generic, Lord et al., 2000). These clinical rating systems used to diagnose ASD based on observation and interpretation of behaviors in a social setting do not include gait, general walking, or any objectively reproducible motor measure from the physical body. Thus there are no existing scores at all to capture such important differences permeating all aspects of social and non-social motor behaviors.

## Discussion

**Figure 11:**
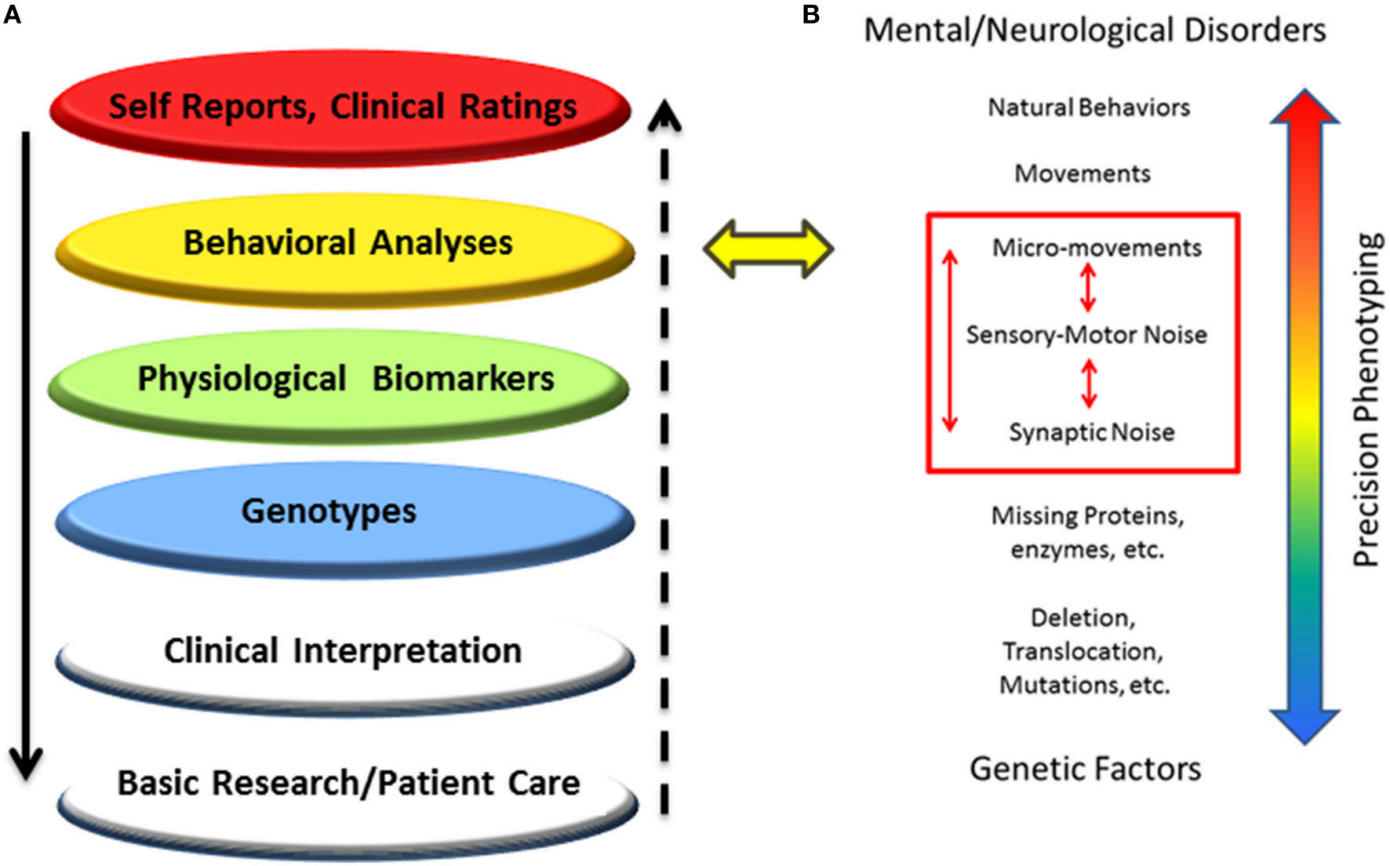
**New proposed methodology to accelerate progress in medicine and science: Schematic to situate the proposed dynamic outcome measures in the network of knowledge for Precision Psychiatry. (A)** Higher layers of outcome data drive basic science and patient care at the bottom layers, but the “evidence-based” outcome of current behavioral analyses prevents personalized medical practices, possibly misinforming bottom layers and negatively impacting decision-making in basic research and patient care. **(B)** The proposed statistical platform to transform current practices into personalized behavioral analyses targeting specific levels of precision involving signatures of motor-sensory based noise with higher likelihood of connecting with genetic factors by exhaustively characterizing different statistical classes of synaptic noise. The layer of behavioral analyses is targeted.

This work characterizes the stochastic signatures of the patterns of micro-movements across the body in children of different ages with PMS. This statistical characterization was performed in relation to controls and also compared to three male individuals with iASD. The latter small group was used to illustrate a highly heterogeneous disorder with three very different individuals with similar clinical diagnoses with the prospect of identifying some statistical pattern common to all three. The results of this characterization underscore the need for individualized assessments of various layers of data to provide a comprehensive phenotype of the person affected by a neurodevelopmental disorder (in this particular case PMS) and better integrate the knowledge from various layers of basic research and clinical practice (Figure 11A).

In all atypical cases, we found excess noise and randomness according to the Fano Factor and other statistical indexes. By examining the stochastic signatures of the moment-by-moment fluctuations in motor performance during walking, we were able to separate levels of spontaneous random noise present in most PMS and iASD participants from well-structured, systematic noise with predictive power in neurotypical controls. In addition to the characterization of signatures of motor noise, we found excess rotational acceleration in PMS. Our interpretation of this feature is that it most likely interferes with their volitional motor control. Excessively accelerated rotations were particularly prevalent in the young 5–7 year old subgroup, and in the adolescents, albeit to a lesser extent. In the iASD individuals, the common hallmark was excess noise and the narrow range of shapes of probability distributions across all 14 joints (*14x3 individuals*, 42 different distributions with similar shape and high dispersion).

Another result came from network analyses. According to the metrics of synchronization that were applied, both iASD and PMS have poor connectivity patterns across the body. These patterns in the older iASD and PMS children and adolescents are comparable to those of a 5-year-old typically developing child. Furthermore, the recruitment/release of degrees of freedom across the upper and lower extremities of the children with neurodevelopmental delays lacked the synergies quantified in controls. The lack of synergies indicates that unlike controls, the nervous systems of the children with PMS and iASD studied here tend to not differentiate the joints of the upper body from those of the lower body in an efficiently articulated manner. Instead, their systems tend to homogeneously recruit all joints across the body. This atypical way of controlling DoF across the body during complex motions is bound to require excess energy; far from the more efficient well-coordinated control strategies detected in all controls. In some of the PMS children, synergies were detected but they did not amount to those quantified in controls. In this sense we were able to statistically characterize the type of poor coordination that is often reported in these populations by observation alone. Moreover, we provide visualization tools to capture the dynamic evolution of the complex network patterns during natural walk, a tool that can serve to track longitudinal performance with great precision.

Analysis of the arm signatures of fluctuations during the walking revealed sex differences in the PMS group, an interesting finding when viewed in the broader context of the ASD diagnosis. As mentioned, all children with PMS also received a formal diagnosis of ASD according to the DSM criteria and the ADOS (see Supplementary Table 1). Perhaps the types of objective analyses introduced here could aid in the clinical phenotyping of ASD in general. One of the advantages of the present methods is that they can be used in the context of any continuous stream of naturalistic motions. Even if the child has communication impairments and cannot follow precise instructions, one could extract the micro-movement patterns from natural motions embedded in bodily rhythms and characterize their statistical signatures as they evolve in time. Using these objective assessments and visualization tools, without constraining the body, it is possible to begin the path of precision phenotyping, thus increasing the likelihood of detecting specific sex features to identify possible ASD subtypes.

### Toward precision psychiatry with proper objective dynamic outcome measures

The results of this work indicate that the proposed biometrics of fluctuations in motor performance may serve as dynamic outcome measures to track bodily rhythms and their evolution over time. The use of these metrics, paired with the advent of high-resolution wearable sensors that register physiological signals, has the potential to accelerate the path toward Precision Psychiatry.

The methods presented are part of a broader statistical platform that aims at an individualized approach to the assessment, tracking, and treatment of disorders of the nervous system in general. In particular, those disorders that result in an atypical neurodevelopmental course would benefit from the use of such dynamic biometrics. We underscore that the current conceptualization of diagnoses and classification of disorders based on observation and coarse clinical ratings has been difficult to connect with the dynamic, non-linear and stochastic nature of neurodevelopment. Any given diagnosis that is treated as a static, deterministic process is bound to become obsolete as soon as the child leaves the clinician's office because the coping nervous system of that child is adapting at a unique rate. This dynamic, non-linear, stochastic process cannot be captured by the “one size fits all” statistical approach currently in use (Torres et al., 2016).

In addition to the methodological statistical issues that traditional methods have, the shifting criteria of the clinical ratings confound the research. Take for example the most recent National Health Interview Survey reported by the CDC on November 13th 2015. By changing the order of the questions asked, among other subjective factors, the estimated annualized prevalence of ASD significantly jumped from 1.25% in the 2011–2013 data to 2.24% in the 2014 data. Unfortunately, at present, scientific research in the areas of neuropsychiatric and neurological disorders is driven by clinical ratings that are just as unreliable. Unlike other areas of medicine, like cancer research, where science leads clinical treatments and patient care, the medical areas addressing disorders of the nervous systems have not made progress toward Precision Medicine (Insel, 2014; Hawgood et al., 2015). Consequently, treatments are symptom-based with no identification of core biological features with the potential to lead toward the discovery of targeted drugs tailored to the person's physiology.

The stochastic signatures of fluctuations on motor performance, as they flow moment by moment, may tap into core biological features of neurodevelopmental disorders such as ASD, and be amenable to constitute one of the dimensions of the RDoC that cuts across research domains (Bernard and Mittal, 2015). Their statistical sensitivity to the subtle rates of change of stochastic signatures, unique to each person, may help us blindly and automatically identify self-emerging clusters across the general iASD population and express them as a function of ASD of known etiology. Conceivably, other groups with a clinical diagnosis of ASD of a known etiology could be studied and similarly characterized. We could then provide a physiologically-relevant statistical map of PDFs (Torres et al., 2016; Figure 7A) with the potential to localize within the general ASD population each self-emerging cluster with a given etiology. This mapping would enable identification of the empirically estimated ranges of sensory-motor noise and randomness levels characteristic of a given group (independent of the clinical phenotype). This is important because muscles across the body serve as natural amplifiers of the underlying synaptic signal flowing across sensory and motor nerves (Kuiken et al., 2009; Hebert et al., 2014). In principle, motor noise depends on synaptic noise (Faisal et al., 2008). In other areas of research, this connection has been successfully exploited at the motor output level to close the sensory motor loops and redirect the information flow of targeted nerves in the periphery so as to implement co-adaptive interfaces for sensory substitution and sensory augmentation (Hebert et al., 2014; Yao et al., 2015). But because ASD is defined exclusively based on behavior, those important fields of neural control of movements and sensory-motor neuroscience that focus on closed-loop neurorehabilitation and sensory-substitution have yet to make contact with the field of autism research at large.

Besides the above mentioned general issues impacting areas of clinical treatments and patient care, there are important methodological considerations for basic research in ASD. The present results from this cohort (Figure 7) and those from other ASD participants in recent work (Torres et al., 2016) point out at the non-Gaussian nature of the distributions of the movement parameters. This indicates that despite their objectivity, typical analytic approaches that average movement parameters across a handful of trials, assume normality, or “detrend” nuisance patterns in the time series of kinematics data of ASD groups, may instead discard biologically relevant signals and provide a mischaracterization of the underlying sensory-motor phenomena. If, in addition to those inadequate statistical practices to asses continuous physiological data, such data are correlated with discrete observational scores (as is common practice today) there is little chance to advance basic research toward Precision Psychiatry.

Besides advantages in research involving human subjects, the use of the proposed biometrics in animal research would be beneficial. At present the phenotype of transgenic models of disorders of the nervous systems (e.g., SHANK3 deletion syndrome or PMS) are descriptive, based on observation, interpretation of the observed behaviors and at best using computerized assessments from hand coded descriptions that are then built into the heuristics of a computer program. Whether by hand or computerized, human heuristics defines discrete aspects of behavior a priori, thus skewing the research to focus on issues that are unambiguous to the naked eye, at the expense of leaving out important spontaneous aspects of behavior that occur largely beneath awareness. Automatic biometric assessment from high grade instrumentation combined with analytics that spontaneously uncover self-emerging patterns from the inherent variability of the data have a chance to accelerate progress toward target treatments and the longitudinal tracking of their effectiveness or risks over different time scales.

### Advancing basic social-science questions in neurodevelopmental disorders with social deficits

The present results speak of a systemic noise problem in disorders that result in a diagnosis of ASD. They extend our previous results in iASD (Torres et al., 2013a,b) to another form of ASD, suggesting a general ***systemic*** micro-movement problem in this population, quantifiable in non-invasive and systematic ways. We have proposed that, paired with this excess sensory-motor noise problem, are issues of sensory-motor integration and sensory-motor transformations that can have a direct impact on socio-motor behavior, deficits that may define the phenotype. For example, lack of proper synergies across the body, such as those revealed here in PMS and iASD, would impede efficient body entrainment during dyadic interactions that take place in social exchange during clinical testing. This could be misinterpreted as lack of joint attention. Yet, in reality, under persistent and systemic random noise across the body, it would be challenging to entrain with any external biological rhythms, such as those present in the social scene during clinical testing. Furthermore, the continuous presence of systemic random noise would prevent any type of proper integration of kinesthetic reafferent (movement-based) feedback with external inputs from biological and non-biological motions coexisting in the environment. In this regard, previous work indicates that typical controls are capable of unambiguously differentiating their own stochastic patterns of walking movements from those of others (Johnson et al., 2012a,b; Mistry et al., 2015). An open question that we can address using the present methods is whether individuals with iASD could also do that, given their reported limitations in understanding biological motions in general (Klin and Jones, 2008; Klin et al., 2009, 2015) and generally preferring non-biological (robotics) motions instead (Giannopulu and Pradel, 2010; Chaminade et al., 2015).

Further investigations into motor sensing patterns may be important in general to advance our understanding of sensory issues in iASD and other neurodevelopmental disorders. Sensory issues have been previously reported (Dinstein et al., 2012) along with various lines of research suggesting synaptic dysfunction (Zoghbi and Bear, 2012; Shcheglovitov et al., 2013) and inhibition/excitation imbalance (Tabuchi et al., 2007; Yizhar et al., 2011; Cochoy et al., 2015) in ASD. We posit that these synaptic disruptions may directly impact synaptic noise, which will be amplified and present within the signatures of motor noise. The structure of motor noise can then be refined to separate spontaneous-random from systematic-predictive patterns using analyses of micro-movements, so as to reveal specific information linked to a genetic condition.

As these minute fluctuations or signatures of motor noise are invisible to the naked eye, the ASD phenomena have been conceptualized as devoid of sensory-motor issues (Lord et al., 2000). Indeed, within the context of Precision Psychiatry, motor issues are not currently considered in the RDoC (Bernard and Mittal, 2015). By conceiving fluctuations in motor performance as a signal embedded in the returning afferent inputs that those self-produced movements themselves cause (von Holst and Mittelstaedt, 1950), we can begin a path of utilizing the body of knowledge from the fields of neural control of movement and applying it to the study of neurodevelopment. This would be particularly useful in the context of IMA (Wolpert and Miall, 1996; Wolpert et al., 1998), the peripheral movement signal informs the central controllers the moment-by-moment accumulation of sensory evidence to predict the sensory consequences of impending decisions and actions (von Holst and Mittelstaedt, 1950; Von Holst, 1954).

Central structures participating in the brain networks for forward prediction, such as those in the cerebellum, are likely to be compromised in PMS (Aldinger et al., 2013; Soorya et al., 2013). Likewise, IMA in iASD are impeded (Gidley Larson et al., 2008; Haswell et al., 2009; Mostofsky and Ewen, 2011), perhaps because important pathways projecting sensory afferents from the body to cerebellar nodes (Mostofsky et al., 2009) and critical nodes in cortical networks (Prevosto et al., 2009, 2010, 2011), relevant in forward prediction (Mulliken et al., 2008; Torres et al., 2013c), are reportedly disrupted in iASD (Gidley Larson et al., 2008; Haswell et al., 2009; Mostofsky et al., 2009; Nebel et al., 2014). Under such brain-body network disruptions it is very unlikely to develop efficient synergies for the volitional control of movement. Even though individuals with ASD move, the moment-by-moment returning motor signal may have narrow bandwidth, and be corrupted by noise and randomness at the periphery (Brincker and Torres, 2013).

We propose that the persistently corrupted kinesthetic motor reafference may have origins at the synapse, with different sub-classes of molecular pathways selectively outputting different motor-noise patterns characterized by specific statistical signatures. This is a testable hypothesis that paired with the tools provided here would guide a new type of precision phenotyping in neurodevelopmental research.

## Conclusions

This study offers a new unifying statistical framework for the personalized analyses of naturalistic behaviors with unprecedented level of detail. We were able to capture and characterize motor phenotypic features of PMS relative to normative data, identify areas of commonality and differences with iASD, and propose a new general statistical platform with the potential to eventually establish a bridge between behavior and genetics at an individualized level (Figures 11A,B). There is a critical need for such a unifying framework and for the type of precision phenotyping introduced here. As such, this work could accelerate the path toward Precision Psychiatry with broad implications across multiple disciplines, including among others behavioral neuroscience, genetics and clinical areas.

## Authors contributions

ET designed the study, analyzed the data and wrote paper; ET, JN, SM, CW, VK, participated in data collection and manuscript preparation; AK provided subject recruitment, clinical supervision and manuscript editing. All authors read and approved the manuscript.

### Conflict of interest statement

The authors declare that the research was conducted in the absence of any commercial or financial relationships that could be construed as a potential conflict of interest. Rutgers The State University of New Jersey and ET hold three provisional US-international patents for the biometrics presented in the paper.

## Abbreviations

PMS: Phelan-McDermid Syndrome
CNS: Central Nervous Systems
PNS: Peripheral Nervous Systems
NPVIndex: Normalized peak velocity index
IMA: Internal Models for Action
PDF: Probability Density Function
eCDF: empirical Cumulative Distribution Function.
