# Supplementary material for: Characterization of the Statistical Signatures of Micro-Movements Underlying Natural Gait Patterns in Children with Phelan McDermid Syndrome: Towards Precision-Phenotyping of Behavior in ASD
Source: Front Integr Neurosci. 2016 Jun 27;10:22. doi: 10.3389/fnint.2016.00022 (PMC4921802; doi:10.3389/fnint.2016.00022)
Supplement: Supplementary file 1 [file Table1.DOCX]

| **Subject** | **Age (yr:mo)** | **The Mullen Scales of Early Learning ^[[1]](#footnote-1)^** | | | **Vineland Adaptive Behavior Scales-II** | | | | | **ADI-R^[[2]](#footnote-2)^** | | | **ADOS-2^[[3]](#footnote-3)^** | | **DSM-IV/ DSM-5** | **Consensus Diagnosis** |
| --- | --- | --- | --- | --- | --- | --- | --- | --- | --- | --- | --- | --- | --- | --- | --- | --- |
|  |  | **Gross Motor** | **Fine Motor** | **NVDQ estimate^[[4]](#footnote-4)^** | **Motor** | **Communication** | **Socialization** | **Daily Living** | **ABC** | **A: Social** | **B: Com** | **C: RRBI** | **SA** | **RRB** |  |  |
| 1 | 5:11 | 18 | 14 | 17.79 | 56 | 42 | 51 | 40 | 45 | 19 | 13 | 1 | 18 | 18 | ASD | ASD |
| 2 | 8:5 | 16 | 13 | 17.81 | 54 | 38 | 48 | 48 | 44 | 23 | 14 | 6 | 19 | 19 | ASD | ASD |
| 3 | 6:4 | 27 | 28 | 21.12 | 61 | 44 | 59 | 51 | 52 | 19 | 10 | 2 | 14 | 14 | ASD | ASD |
| 4 | 5:11 | - | 22 | 27.46 | 59 | 49 | 57 | 48 | 51 | 11 | 13 | 3 | 7 | 7 | ASD | ASD |
| 5 | 6:1 | 18 | 9 | 13.69 | 54 | 36 | 48 | 34 | 41 | 28 | 14 | 7 | 17 | 17 | ASD | ASD |
| 6 | 7:10 | 20 | 15 | 15.43 | 49 | 45 | 48 | 51 | 48 | 30 | 14 | 6 | 20 | 20 | ASD | ASD |
| 7 | 7:8 | 33 | 33 | 8.68 | 56 | 45 | 53 | 51 | 50 | 23 | 13 | 6 | 16 | 16 | ASD | ASD |
| 8 | 9:1 | 13 | 15 | 17.094 | 81 | 54 | 66 | 63 | 61 | 5 | 6 | 4 | 15 | 15 | ASD | ASD |
| 9 | 14:8 | 29 | 28 | 19.39 | 56 | 35 | 32 | 35 | 31 | 16 | 12 | 8 | 20 | 20 | ASD | ASD |
| 10 | 5:11 | 22 | 10 | 9.86 | 56 | 36 | 51 | 38 | 43 | 24 | 12 | 5 | 18 | 18 | ASD | ASD |
| 11 | 15:9 | - | 6 | 4.22 | 56 | 33 | 37 | 28 | 29 | 28 | 14 | 2 | 17 | 17 | ASD | ASD |
| 12 | 5:0 | 14 | 6 | 26.67 | 43 | 40 | 51 | 41 | 43 | 25 | 14 | 8 | 4 | 14 | ASD | ASD |
| 13 | 8:7 | 15 | 13 | 12.01 | 43 | 47 | 48 | 48 | 47 | 28 | 14 | 2 | 5 | 10 | ASD | ASD |
| 14 | 5:9 | 22 | 21 | 34.06 | 54 | 61 | 61 | 55 | 55 | 13 | 15 | 0 | 16 | 4 | ASD | ASD |
| 15 | 5:1 | 21 | 17 | 36.07 | 56 | 52 | 51 | 43 | 49 | 23 | 14 | 6 | 20 | 5 | ASD | ASD |
| 16 | 12:6 | 30 | 20 | 13.51 | 64 | 42 | 42 | 50 | 44 | 29 | 13 | 4 | 22 | 3 | ASD | ASD |

| **Subject** | **Chronological age**  **(years)** | **Sex** | **Subject Type** |
| --- | --- | --- | --- |
| 17 | 12 | M | Idiopathic ASD |
| 18 | 11 | M | Idiopathic ASD |
| 19 | 10 | M | Idiopathic ASD |
| NEUROTYPICAL CONTROLS | | | |
| 20 | 5 | F | Neurotypical |
| 21 | 6 | F | Neurotypical |
| 22 | 7 | F | Neurotypical |
| 23 | 8 | M | Neurotypical |
| 24 | 13 | M | Neurotypical |
| 25 | 7 | M | Neurotypical |
| 26 | 16 | F | Neurotypical |
| 27 | 19 | M | Neurotypical - Athlete |
| 28 | 18 | F | Neurotypical |
| 29 | 16 | M | Neurotypical |
| 30 | 17 | M | Neurotypical |

**Supplementary Tables 1-2**: Participant’s demographic and clinical information.

1. The Mullen Scales of Early Learning; Gross Motor and Fine Motor scores provided are the age equivalent in months [↑](#footnote-ref-1)
2. ADI-R cutoff scores for autism are: Social = 10, Communication (nonverbal) = 7, Repetitive Behaviors and Restricted Interests = 3 [↑](#footnote-ref-2)
3. All participants received a Toddler Module or Module 1 of the ADOS-2

   Abbreviations: ABC, Adaptive Behavior Composite (Vineland); ADI-R, Autism Diagnostic Interview-Revised; A: Social, Qualitative Abnormalities in Reciprocal Social Interaction; B: Com, Communication; C: Repetitive and Restricted Behaviors; ADOS, Autism Diagnostic Observation Schedule; Com Total, ADOS-2 SA, Social Affect Total, ADOS-2 RRB, Restricted and Repetitive Behavior Total; NVDQ, Nonverbal Developmental Quotient [↑](#footnote-ref-3)
4. The Mullen Scales of Early Learning; DVIQ scores were calculated by averaging the age equivalent for fine motor and visual reception subtests then dividing by the child's age in months and multiplying by 100 (Bishop, Guthrie, Coffing, & Lord, 2011) [↑](#footnote-ref-4)
