# Supplementary material for: Characterization of the Statistical Signatures of Micro-Movements Underlying Natural Gait Patterns in Children with Phelan McDermid Syndrome: Towards Precision-Phenotyping of Behavior in ASD
Source: Front Integr Neurosci. 2016 Jun 27;10:22. doi: 10.3389/fnint.2016.00022 (PMC4921802; doi:10.3389/fnint.2016.00022)
Supplement: Supplementary file 2 [file DataSheet1.docx]

# Supplementary Figures


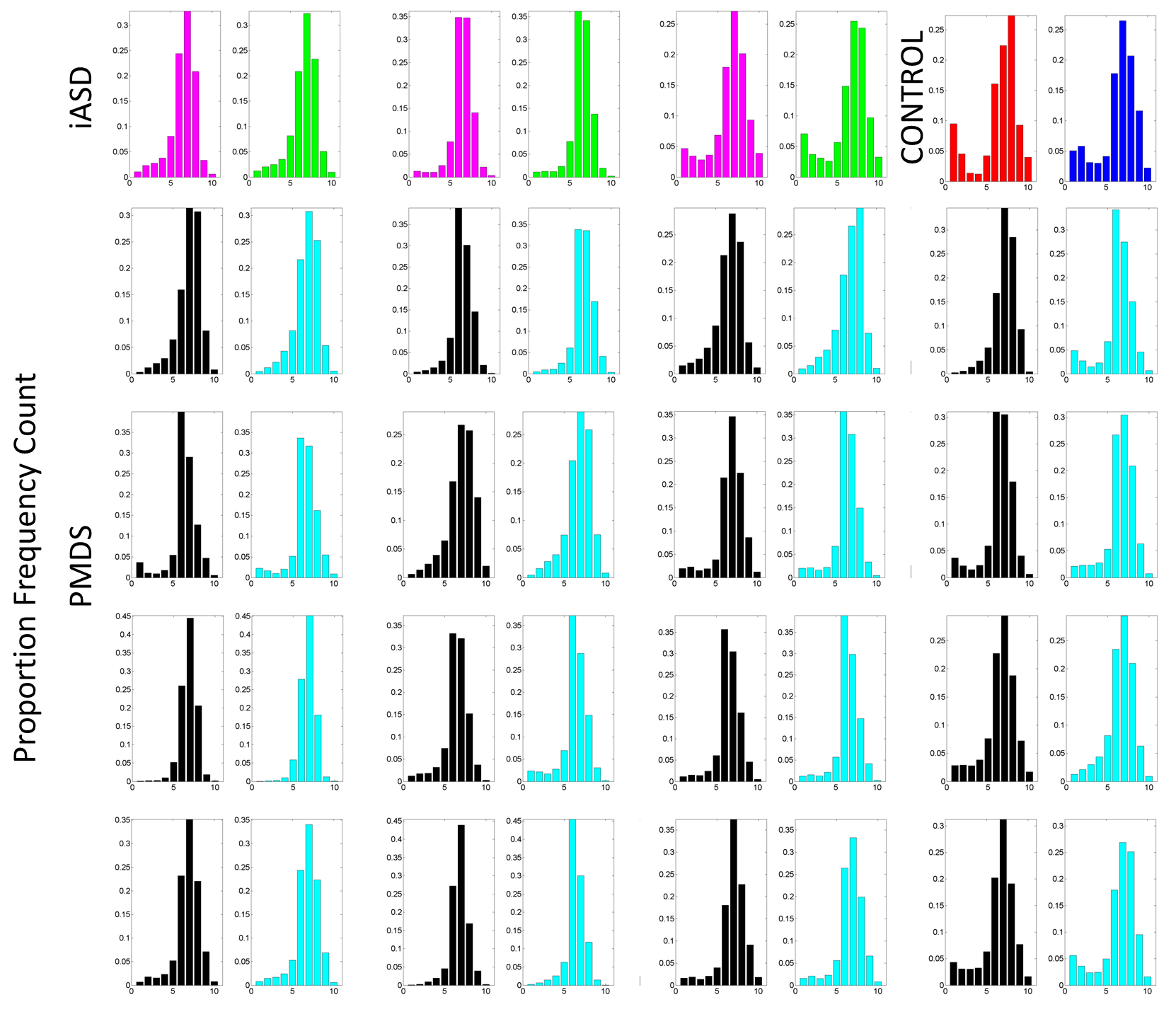


**Supplementary Figure 1**. Barplots of the proportion of count of actual angular velocity peaks for representative children in each group under study complementing Figure 6 of the main text (containing the histograms of the normalized peak velocity index, normalized to account for possible allometry effects.)


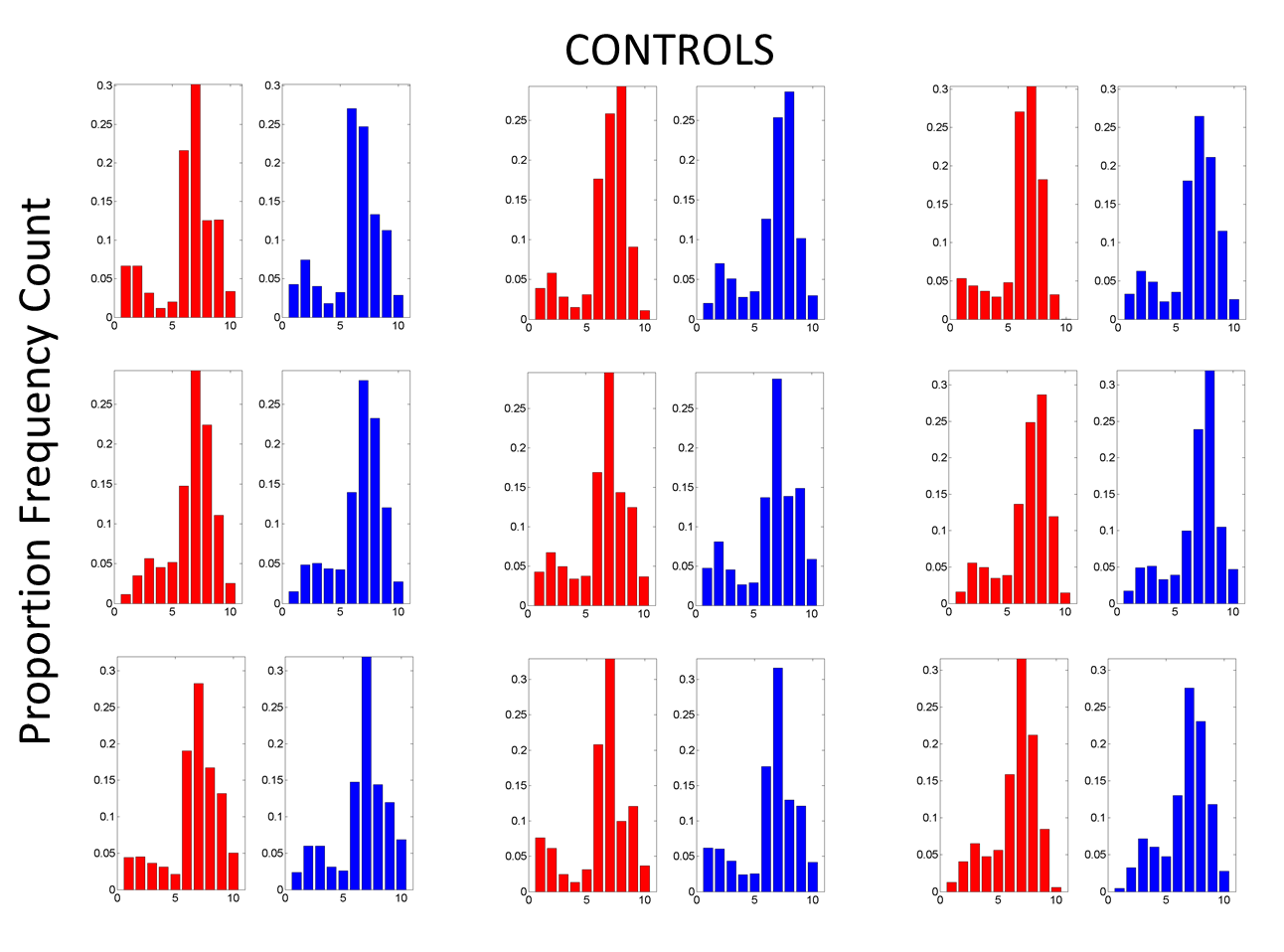


**Supplementary Figure 2**. Barplots of the proportion of count of actual angular velocity peaks for representative typical children and adolescents complementing those in Figure 6 of the main text (containing the histograms of the normalized peak velocity index, normalized to account for possible allometry effects.) Notice the multiple bumps in the distributions absent in most PMS children (who have either a total absence of multimodality in both feet or a modest additional bump in one foot). No PMS has significant evidence of multimodality in both feet.


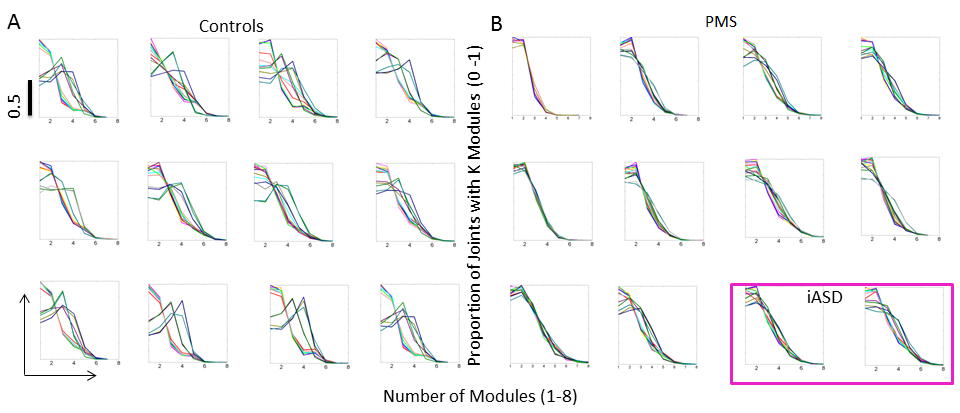


**Supplementary Figure 3**. Additional examples of bodily synergies uncovered in controls that are absent from the motions of the PMS children and from those of 2/3 children with iASD. Horizontal axis is the number of modules across the network of 14 joints. Vertical axis is the proportion of joints with K modules (ranging from 0 to 1).


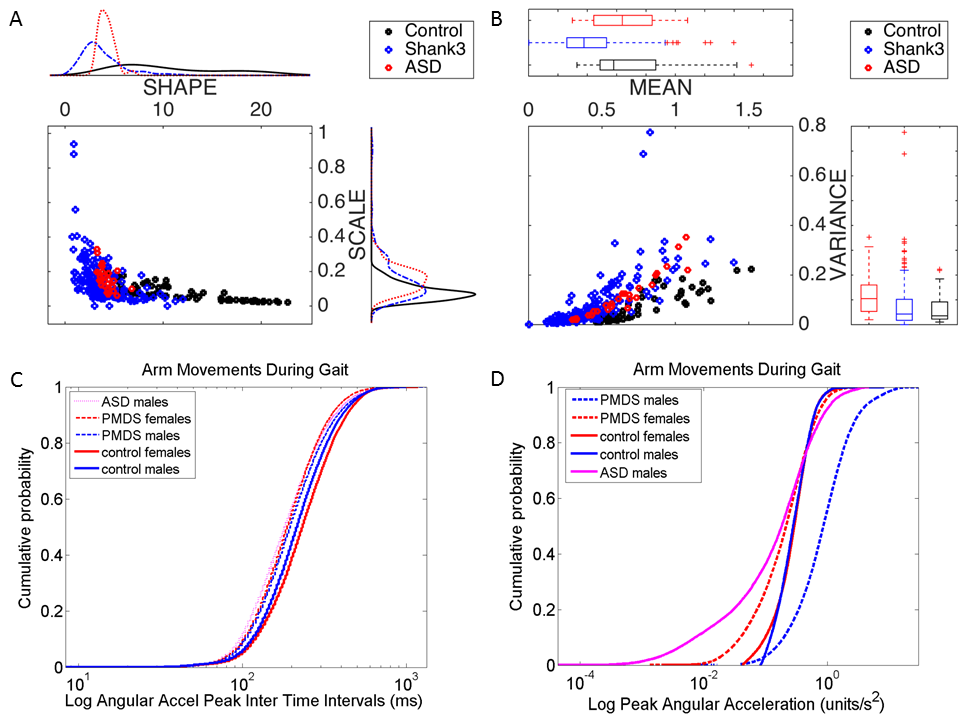


**Supplementary Figure 4**. Additional examples of atypical stochastic signatures in PMS and iASD. (A) Distributions of the empirically estimated shape and scale parameters of the continuous Gamma family of PDFs across the 14 joints of the body using the micro-movements embedded in the angular accelerations. (B) Scatters of estimated first (mean) and second (variance) moments of the Gamma PDFs using the estimated shape and scale values in (A) and statistical comparisons using non-parametric ANOVA reveal statistically significant differences at the alpha 0.01 level. (C) Differences in the timing of the peak angular acceleration captured in the cumulative distribution function across subject types. (D) Differences in patterns of angular acceleration micro-movements captured by the cumulative distribution functions across subject types (see legends).
